# Intracellular Ser/Thr/Tyr phosphoproteome of the oral commensal *Streptococcus gordonii* DL1

**DOI:** 10.1186/s12866-020-01944-y

**Published:** 2020-09-14

**Authors:** Carolina Robertsson, Gunnel Svensäter, Zoltan Blum, Claes Wickström

**Affiliations:** 1 Department of Oral Biology and Pathology, Faculty of Odontology, Malmö University, 20506 Malmö, Sweden; 2 Department of Biomedical Science, Malmö University, 20506 Malmö, Sweden

**Keywords:** 2DE, Oral bacteria, Phosphoproteome, Pro-Q diamond, Streptococci, *Streptococcus gordonii*

## Abstract

**Background:**

To respond and adapt to environmental challenges, prokaryotes regulate cellular processes rapidly and reversibly through protein phosphorylation and dephosphorylation. This study investigates the intracellular proteome and Ser/Thr/Tyr phosphoproteome of the oral commensal *Streptococcus gordonii*. Intracellular proteins from planktonic cells of *S. gordonii* DL1 were extracted and subjected to 2D-gel electrophoresis. Proteins in general were visualized using Coomassie Brilliant Blue and T-Rex staining. Phosphorylated proteins were visualized with Pro-Q Diamond Phosphoprotein Gel Stain. Proteins were identified by LC-MS/MS and sequence analysis.

**Results:**

In total, sixty-one intracellular proteins were identified in *S. gordonii* DL1, many of which occurred at multiple isoelectric points. Nineteen of these proteins were present as one or more Ser/Thr/Tyr phosphorylated form. The identified phosphoproteins turned out to be involved in a variety of cellular processes.

**Conclusion:**

Nineteen phosphoproteins involved in various cellular functions were identified in *S. gordonii*. This is the first time the global intracellular Ser/Thr/Tyr phosphorylation profile has been analysed in an oral streptococcus. Comparison with phosphoproteomes of other species from previous studies showed many similarities. Proteins that are consistently found in a phosphorylated state across several species and growth conditions may represent a core phosphoproteome profile shared by many bacteria.

## Background

*Streptococcus gordonii* is a pioneer oral colonizer, involved in the establishment of oral biofilms [1]. Due to the numerous different adhesins expressed on its surface, *S. gordonii* cells readily attach to and colonize dental surfaces [2]. Moreover, *S. gordonii* can passively migrate from small oral lesions through the blood stream and cause infective endocarditis by opportunistic infection of the heart valves [3]. This mainly saccharolytic species is considered a commensal with ubiquitous habitation in humans, and given the acid production and acid tolerance of *S. gordonii*, it prevails at the acidic conditions that periodically occur in oral biofilms [4–6]. When carbohydrate concentrations are low, *S. gordonii* like other oral streptococci utilizes a carbohydrate phosphotransferase transport system (PTS) with high glucose affinity [7]. Upon spikes in carbohydrate concentration, oral streptococci are at risk for “sugar killing” from damaging effects caused by accumulated glycolytic intermediates [8]. To evade such inflictions, oral streptococci switch to carbohydrate transport systems with lower affinity for glucose, *e.g.* the permease system. The glycolytic rate can also be regulated by switching to alternative pathways, *e.g.* through activation of lactate dehydrogenase (ldh) in pyruvate conversion for faster regeneration of NAD^+^ [9]. In this way, cells reduce glucose uptake as well as drain themselves of glycolytic intermediates by producing large amounts of lactic acid. In addition, oral streptococci employ strategies colloquially termed as the “acid tolerance response” (ATR) that enhance cell survival in acidic environments [4, 10, 11]. The ATR is dependent on molecular chaperone activity [4, 8] that sustains correct protein folding during biosynthesis even at low pH.

To keep up with environmental fluctuations, prokaryotes have been suggested to regulate the activity of proteins involved in the central carbon metabolism rapidly and reversibly through phosphorylation and dephosphorylation mechanisms [12, 13]. The reversible regulatory phosphorylation events operate on a much faster time scale than changes in protein expression [14]. The earliest studies on protein phosphorylation and dephosphorylation as regulatory events in bacteria focused on phosphorylation of histidine and aspartate residues in relation to two-component systems [15]. However, phosphorylation events on serine, threonine, and tyrosine (Ser/Thr/Tyr) have also been found to play important roles in prokaryotic intracellular signalling. Although the most common group of Ser/Thr kinases (i.e. Hanks-type kinases) are often referred to as “eukaryotic kinases”, many prokaryotic Ser/Thr kinases also belong to this group [16]. There are many examples of phosphorylation events regulating cellular activities in bacteria, relating to house-keeping functions as well as stress responses and virulence [12, 17, 18]. In *Lactobacillus rhamnosus,* phosphorylation of glycolytic enzymes is upregulated as a response to acidic stress [19]. In that study, the phosphorylation state of threonine and serine residues on glyceraldehyde 3-phosphate dehydrogenase (gapdh) differed between protein species depending on the growth conditions, while the overall abundance of the different proteins remained unchanged. This supports the idea that phosphorylation can regulate enzyme activity separately from regulation of gene expression. Different forms of the same protein may display increased or decreased catalytic activity, altered subcellular localization, or modified interaction with non-substrates [20, 21]. Multisite phosphorylation can coordinate several such effects, determine the duration of a response, or mediate signal integration [21]. Protein phosphorylation may also completely alter the biological functions of proteins in the cell, a phenomenon referred to as protein moonlighting [22]. The regulatory effects of phosphorylation events on specific enzymes have been studied in some oral bacteria, mainly the oral streptococci [23–25], but to the best of our knowledge, global Ser/Thr/Tyr phosphorylation profiles of *S. gordonii* have not yet been detailed. The aim of the current study was to identify the intracellular protein expression profile, with special attention to Ser/Thr/Tyr phosphorylated proteins, in *S. gordonii* DL1.

## Results

### General protein expression profile

**Fig. 1:**
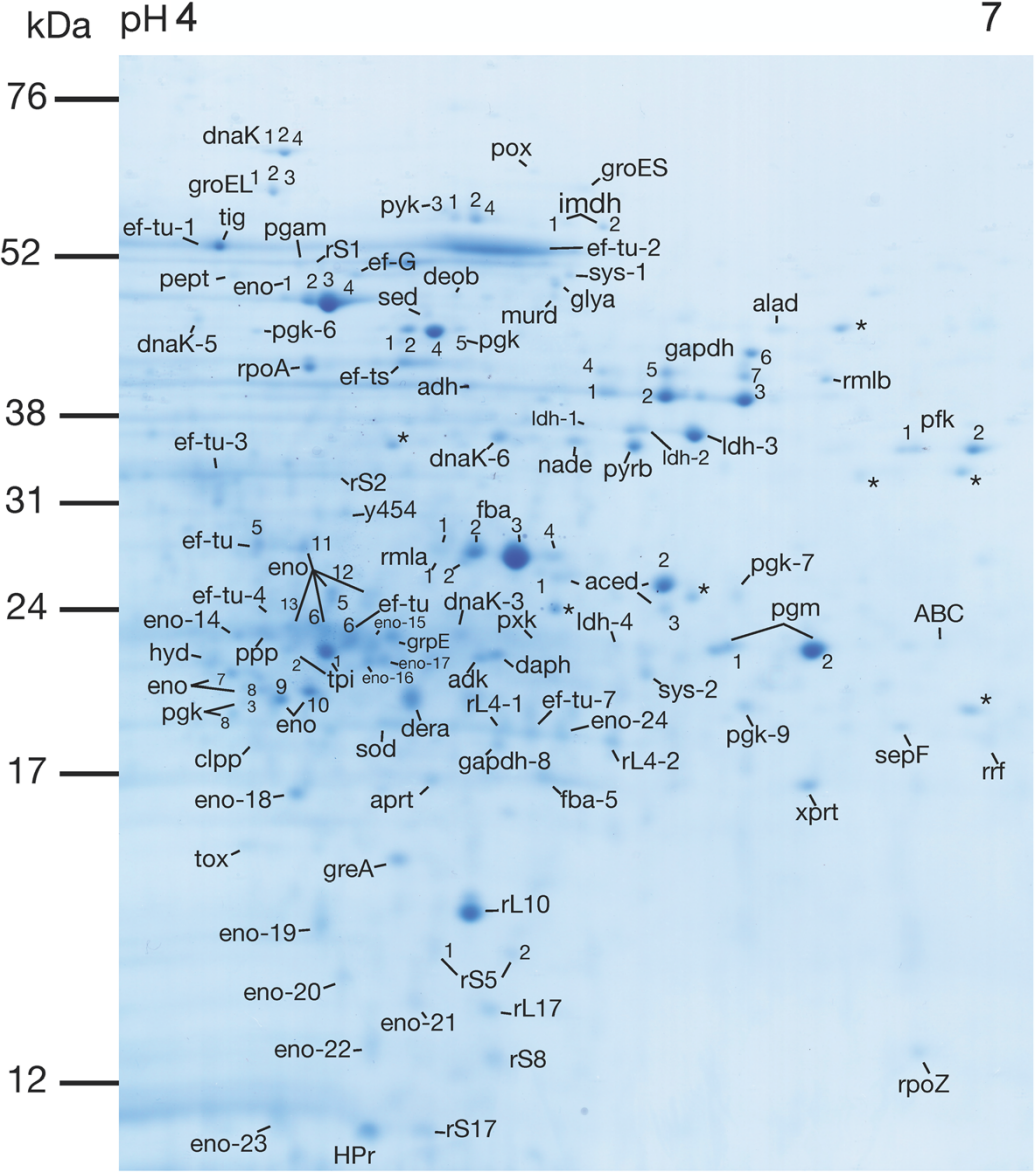
Representative 2DE protein expression profile extracted from *S. gordonii* DL1, visualized with Coomassie Brilliant Blue stain. Proteins identified by mass spectrometry are indicated (abbreviations are listed in Table 1). Spots with no significant hits in the Mascot database search are labelled with an asterisk (*). Gels were produced in triplicates from three different cultures of *S. gordonii* DL1

Intracellular proteomes from planktonic cells of *S. gordonii* DL1 were extracted and separated by 2DE. The total intracellular proteome was visualized with Coomassie Brilliant Blue stain (Sigma). In total, 222 protein spots were detected. Discrete spots were manually excised for identification with LC-MS/MS. Molecular weights (MWs) and isoelectric points (p*I*s) for identified proteins were estimated from the gels as well as gathered from the mass spectrometry data. MWs, p*I*s, MASCOT scores, number of matched peptides and % coverages are listed in Table 1. The sequenced peptides identified by LC-MS/MS can be found in supplemental material (Additional file 1). In total, 61 proteins were identified, many of which were present at multiple isoelectric points (Fig. 1, Table 1).

**Table 1:** *S. gordonii* intracellular proteins and phosphoproteins, separated by 2DE and identified with LC-MS/MS

Abbr	Protein	Gene^b)^	Acc. No.^c)^	pI estimated from gel/theoretical^d)^	MW, Da estimated from gel/ theoretical^d)^	LC-MS/MS			Ser/Thr/Tyr phosphory-lated +/−
						MASCOT score	No. of peptides^e)^	Coverage	
**Transmembrane transport**									
ABC^a)^	ABC transporter ATP-binding protein SP1715	SGO_1342	A8AXW3	6.7/6.25	23,000/26325	1124	18	72%	–
**Sugar transport**									
HPr^a)^	Phosphocarrier protein HPr	*ptsH* (SGO_1556)	A8AYH5	5.1/4.74	11,000/8935	124	2	55%	+
**Glycolysis**									
Tpi-1^a)^	Triosephosphate isomerase	*tpiA (SGO_0762)*	A8AWA1	4.5/4.75	23,000/26524	652	9	52%	+
Tpi-2^a)^				4.3/4.75	23,000/26524	540	9	52%	-
pfk-1^a)^	Phosphofructokinase	*pfkA (SGO_1340)*	A8AXW1	6.6/5.62	35,000/35346	1043	17	49%	+
pfk-2				6.8/5.62	35,000/35346	1476	22	56%	+
fba-1^f)^	Fructose-1,6-bisphosphate aldolase, class II	*Fba (SGO_1745)*	A8AZ06	5.1/5.00	27,000/31394	-	-	-	-
fba-2^a)^				5.2/5.00	27,000/31394	771	12	51%	-
fba-3^a)^				5.3/5.00	27,000/31394	800	14	47%	-
fba-4^f)^				5.4/5.00	27,000/31394	-	-	-	-
fba-5^a)^				5.4/5.00	17,000/31394	164	3	11%	-
gapdh-1^a)^	Glyceraldehyde-3-phosphate dehydrogenase	*gap (SGO_0207)*	A8AUR7	5.5/5.37	39,000/35918	1153	20	51%	+
gapdh-2^a)^				5.7/5.37	39,000/35918	1299	21	79%	+
gapdh-3^a)^				5.9/5.37	39,000/35918	1304	22	58%	+
gapdh-4				5.5/5.37	40,000/35918	89	2	8%	-
gapdh-5				5.7/5.37	40,000/35918	276	4	14%	-
gapdh-6				6.0/5.37	39,000/35918	84	2	6%	-
gapdh-7				5.9/5.37	40,000/35918	325	5	16%	-
gapdh-8				5.2/5.37	18,000/35918	141	2	8%	-
pgk-1	Phosphoglycerate kinase	*pgk (SGO_0209)*	A8AUR9	4.9/4.88	45,000/42089	397	6	18%	+
pgk -2^a)^				5.0/4.88	45,000/42089	1454	23	63%	+
pgk -3^a)^				4.3/4.88	21,000/42089	340	5	17%	+
pgk -4^f)^				5.1/4.88	45,000/42089	-	-	-	-
pgk -5^f)^				5.2/4.88	45,000/42089	-	-	-	-
pgk −6				4.3/4.88	45,000/42089	159	3	9%	-
pgk -7^a)^				5.9/4.88	24,000/42089	431	7	18%	-
pgk -8^a)^				4.2/4.88	20,000/42089	830	11	41%	-
pgk -9^a)^				5.9/4.88	20,000/42089	561	9	28%	-
pgm-1^a)^	Phosphoglycerate mutase	*gpma (SGO_0704)*	A8AW46	5.9/5.41	22,000/26044	904	18	84%	+
pgm-2^a)^				6.1/5.41	22,000/26044	933	18	87%	+
eno-1	Enolase	*eno (SGO_1426)*	A8AY46	4.4/4.71	48,000/47062	1368	18	61%	+
eno −2				4.5/4.71	48,000/47062	1260	13	52%	+
eno −3				4.6/4.71	48,000/47062	1721	18	63%	+
eno -4^a)^				4.7/4.71	48,000/47062	2245	31	77%	+
eno −5				4.6/4.71	26,000/47062	1316	17	58%	+
eno -6^a)^				4.5/4.71	24,000/47062	460	8	26%	+
eno -7^a)^				4.1/4.71	22,000/47062	809	9	30%	+
eno -8^a)^				4.2/4.71	21,000/47062	673	9	28%	+
eno -9^a)^				4.3/4.71	21,000/47062	595	10	32%	+
eno -10^a)^				4.4/4.71	21,000/47062	989	11	34%	+
eno -11^a)^				4.4/4.71	28,000/47062	1485	15	55%	-
eno -12^a)^				4.8/4.71	25,000/47062	950	14	47%	-
eno -13^a)^				4.4/4.71	23,000/47062	460	8	26%	-
eno -14^a)^				4.2/4.71	23,000/47062	746	11	36%	-
eno -15^a)^				4.8/4.71	23,000/47062	627	9	32%	-
eno -16^a)^				4.8/4.71	22,000/47062	631	9	31%	-
eno -17^a)^				4.9/4.71	22,000/47062	636	9	31%	-
eno -18^a)^				4.4/4.71	17,000/47062	230	4	11%	-
eno −19				4.5/4.71	14,500/47062	931	9	41%	-
eno − 20				4.6/4.71	14,000/47062	533	7	23%	-
eno -21^a)^				5.0/4.71	13,000/47062	437	7	23%	-
eno -22^a)^				4.7/4.71	12,500/47062	722	9	33%	-
eno -23^a)^				4.3/4.71	11,000/47062	418	6	20%	-
eno -24^a)^				5.4/4.71	18,000/47062	684	9	27%	-
pyk-1^a)^	Pyruvate kinase	*pyk (SGO_1339)*	A8AXW0	5.15/4.94	58,000/54799	150	3	6%	+
pyk-2^a)^				5.2/4.94	58,000/54799	1474	29	62%	+
pyk-3^f)^				5.1/4.94	58,000/54799	-	-	-	-
pyk-4^f)^				5.3/4.94	58,000/54799	-	-	-	-
**Cofactor biosynthesis**									
nade^a)^	NH(3)-dependent NAD(+) synthetase	*nadE* (SGO_0583)	A8AVT9	5.4/5.11	35,000/30248	268	3	16%	–
**Acetoin catabolism**									
aced-1	Acetoin dehydrogenase	*butA (SGO_1096)*	A8AX75	5.4/5.20	26,000/26547	1008	15	77%	-
aced-2^a)^				5.7/5.20	26,000/26547	1247	17	75%	-
aced-3^a)^				5.7/5.20	24,000/26547	809	13	62%	-
**Carbohydrate catabolism**									
dera^a)^	Deoxyribose-phosphate aldolase	*deoC* (SGO_1080)	A8AX59	4.9/4.84	20,000/23380	691	8	49%	–
**Pyruvate conversion**									
ldh-1^a)^	L-lactate dehydrogenase	*ldh (SGO_1232)*	A8AXK9	5.4/5.24	36,000/35267	327	6	28%	+
ldh-2				5.6/5.24	36,000/35267	436	6	28%	+
ldh-3				5.8/5.24	36,000/35267	641	8	32%	-
adh^a)^	Alcohol dehydrogenase	*adhA* (SGO_0565)	A8AVS1	5.2/4.94	39,000/35958	911	13	69%	–
pox	Pyruvate oxidase	*spxB* (SGO_0292)	A8AV01	5.3/5.06	65,000/65283	161	3	5%	–
**Pentose phosphate pathway**									
deob	Phosphopentomutase	*deoB* (SGO_1264)	A8AXN8	5.3/4.96	47,000/44508	172	3	9%	+
**Amino acid metabolism**									
alad	Alanine dehydrogenase	*ald* (SGO_0708)	A8AW50	6.1/5.29	45,000/38960	1378	19	63%	–
daph^a)^	2,3,4,5-tetrahydropyridine-2,6-dicarboxylate N-acetyltransferase	*dapH* (SGO_0158)	A8AUL9	5.2/5.06	22,000/24255	291	5	25%	–
glya	Serine hydroxymethyltransferase	*glyA,* (SGO_1151)	A8AXC8	5.4/5.07	48,000/45566	583	8	28%	–
sed	Homoserine dehydrogenase	*hom* (SGO_0801)	A8AWE0	5.0/4.86	45,000/46154	245	3	11%	–
**Nucleotide metabolism**									
adk^a)^	Adenylate kinase	*adk* (SGO_1964)	A8AZK4	5.2/4.95	22,000/ 23,857	318	6	38%	–
aprt^a)^	Adenine phosphoribosyltransferase	*apt* (SGO_1001)	A8AWY0	5.0/4.90	17,000/18840	875	12	74%	–
imdh-1	Inosine-5′-monophosphate dehydrogenase	*guaB (SGO_0008)*	A8AU70	5.4/5.19	58,000/52717	116	2	5%	+
imdh-2				5.5/5.19	56,000/52717	252	4	12%	-
pyrb	Aspartate carbamoyltransferase	*pyrB* (SGO_1109)	A8AX88	5.6/5.04	35,000/34741	54	1^g)^	4%	–
xprt	Xanthine phosphoribosyltransferase	*xpt* (SGO_1158)	A8AXD5	6.2/5.79	17,000/20849	684	12	77%	–
**Molecular chaperone activity and/or protein folding**									
Dnak-1	Molecular chaperone DnaK	*dnaK (SGO_0402)*	A8AVA8	4.4/4.69	68,000/64724	266	4	9%	+
DnaK-2^a)^				4.5/4.69	68,000/64724	1557	26	50%	+
DnaK-3^a)^				5.2/4.69	25,000/64724	134	2	3%	+
DnaK-4^f)^				4.6/4.69	68,000/64724	-	-	-	-
DnaK-5				4.1/4.69	44,000/64724	254	4	10%	-
DnaK-6^a)^				5.3/4.69	36,000/64724	345	6	14%	-
GroEL-1	Molecular chaperone GroEL	*groEL (SGO_1885)*	A8AZE1	4.2/4.66	64,000/56754	776	13	34%	+
GroEL-2				4.3/4.66	64,000/56754	1500	22	47%	+
GroEL-3				4.4/4.66	64,000/56754	794	13	32%	-
GroES	Co-chaperone GroES	*groES* (SGO_1886)	A8AZE2	5.4/5.11	64,000/9681	65	1^g)^	2%	–
grpE^a)^	Molecular chaperone GrpE	*grpE* (SGO_0401)	A8AVA7	4.8/4.83	23,000/20265	186	3	18%	–
hyd^a)^	Hydrolase, haloacid dehalogenase family/ peptidyl-prolyl cis-trans isomerase, cyclophilin type	SGO_0604	A8AVW0	4.2/4.74	22,000/51936	516	10	30%	–
tig^a)^	Trigger factor (chaperone)	*tig* (SGO_0412)	A8AVB8	4.2/4.53	55,000/47242	531	10	31%	+
**Transcription**									
GreA	Transcription elongation factor GreA	*greA* (SGO_0519)	A8AVM5	4.9/4.85	15,500/17515	581	9	68%	–
rpoA^a)^	DNA-directed RNA polymerase subunit alpha	*rpoA* (SGO_1959)	A8AZJ9	4.5/4.70	41,000/34502	578	7	29%	–
rpoZ	DNA-directed RNA polymerase subunit omega	*rpoZ* (SGO_0595)	A8AVV1	6.7/5.82	12,000/11851	231	5	44%	–
y454	Probable transcriptional regulatory protein		A8AVG0	4.6/4.45	30,000/25722	227	4	26%	–
**Translation**									
ef-G	Elongation factor G	*fusA* (SGO_0206)	A8AUR6	4.7/4.88	50,000/76785	53	1^g)^	2%	–
ef-Ts	Elongation factor Ts	*tsf* (SGO_2000)	A8AZP0	4.9/4.84	41,000/37284	326	5	20%	–
ef-tu-1^a)^	Elongation factor tu	*tuf (SGO_0761)*	A8AWA0	4.1/4.86	55,000/44011	519	8	31%	+
ef-tu-2				5–6/4.86	55,000/44011	1253	22	63%	+
ef-tu-3^a)^				4.2/4.86	34,000/44011	532	6	23%	+
ef-tu-4^a)^				4.3/4.86	25,000/44011	167	3	8%	+
ef-tu-5^a)^				4.3/4.86	29,000/44011	553	6	25%	-
ef-tu-6^a)^				4.7/4.86	23,000/44011	420	6	18%	-
ef-tu-7^a)^				5.3/4.86	20,000/44011	433	6	16%	-
rL1^a)^	50S ribosomal protein L1	*rplA* (SGO_1455)	A8AY74	5.5/9.22	23,000/24399	202	3	16%	–
rL4-1^a)^	50S ribosomal protein L4	*rplD (SGO_1984)*	A8AZM4	5.2/10.03	19,000/22279	285	4	26%	-
rL4-2^a)^				5.5/10.03	19,000/22279	327	4	24%	-
rL10^a)^	50S ribosomal protein L10	*rplJ* (SGO_1192)	A8AXG9	5.2/5.06	15,000/17462	1002	15	86%	–
rL17	50S ribosomal protein L17	*rplQ* (SGO_1958)	A8AZJ8	5.3/9.86	13,000/14511	141	2	17%	–
rrf^a)^	Ribosome recycling factor	*frr* (SGO_1451)	A8AY70	6.8/5.90	18,000/20640	421	8	46%	–
rS1	30S ribosomal protein S1	*rpsA* (SGO_1234)	A8AXL0	4.5/4.91	52,000/44149	130	2	5%	+
rS2	30S ribosomal protein S2	*rpsB* (SGO_2001)	A8AZP1	4.6/5.07	33,000/29125	515	8	35%	–
rS5-1^a)^	30S ribosomal protein S5	*rpsE (SGO_1968)*	A8AZK8	5.1/9.69	14,000/17079	335	4	33%	-
rS5–2				5.3/9.69	14,000/17079	283	4	33%	-
rS8^a)^	30S ribosomal protein S8	*rpsH* (SGO_1971)	A8AZL1	5.3/9.48	12,500/14753	120	2	25%	–
rS17	30S ribosomal protein S17	*rpsQ* (SGO_1976)	A8AZL6	5.0/9.92	11,000/10010	58	1^g)^	10%	–
sys-1	Serine tRNA-ligase	*serS (SGO_1683)*	A8AYV0	5.4/5.11	48,000/48110	342	7	21%	-
sys-2^a)^				5.7/5.11	21,000/48110	95	1^g)^	2%	-
**Protein catabolism**									
clpp	ATP-dependent Clp protease proteolytic subunit	*clpP* (SGO_1632)	A8AYP9	4.3/4.72	18,000/21363	200	3	12%	–
pept	Peptidase T	*pepT* (SGO_1312)	A8AXT3	4.2/4.59	49,000/45200	332	4	13%	–
**Vitamin B biosynthesis**									
pxk^a)^	Pyridoxine kinase	SGO_0409	A8AVB5	5.3/5.16	23,000/27598	583	10	52%	–
**Cell envelope biosynthesis**									
murd^a)^	UDP-N-acetylmuramoylalanine D-glutamate ligase	*murdD* (SGO_0671)	A8AW14	5.4/5.07	46,000/48257	253	4	12%	–
pgam^a)^	Phosphoglucosamine mutase	*glmM* (SGO_0889)	A8AWM5	4.6/4.71	52,000/48396	592	7	26%	+
rmla-1	Glucose-1-phosphate thymidylyltransferase	*rfbA-1 (SGO_1009)*	A8AWY8	5.1/4.92	28,000/32213	42	1^g)^	3%	+
rmla-2^a)^				5.3/4.92	28,000/32213	136	2	7%	+
rmlb	dTDP-glucose 4,6-dehydratase	*rfbB-1* (SGO_1011)	A8AWZ0	6.3/5.55	40,000/39314	168	3	12%	–
**Cell division**									
SepF	Cell division protein SepF	*sepF* (SGO_0677)	A8AW20	6.6/5.61	19,000/21654	367	6	41%	–
**Antioxidant activity**									
sod^a)^	Superoxide dismutase	*sodA* (SGO_1599)	A8AYL7	4.9/4.78	19,000/22446	474	8	46%	–
tox	Thiol peroxidase	*Tpx* (SGO_1803)	A8AZ62	4.2/4.52	16,000/18015	349	6	59%	–
**Protein dephosphorylation**									
ppp^a)^	Phosphoprotein phosphatase SGO_0599	SGO_0599	A8AVV5	4.3/4.62	23,000/26870	696	11	61%	+

a) Two or more proteins were identified from this spot

b) Gene name in the UniProt database as entered for *S. gordonii* DL1 proteins

c) Accession number in the UniProt database for *S. gordonii* DL1

d) Theoretical values for *S. gordonii* DL1 proteins

e) The sequenced peptides identified by LC-MS/MS can be found in supplemental material (Additional file 1)

f) Protein identity deduced from adjacent identified spot

g) Proteins identified by only one peptide are given when only a single match was yielded from the database search

### Intracellular Ser/Thr/Tyr phosphorylation profile

**Fig. 2:**
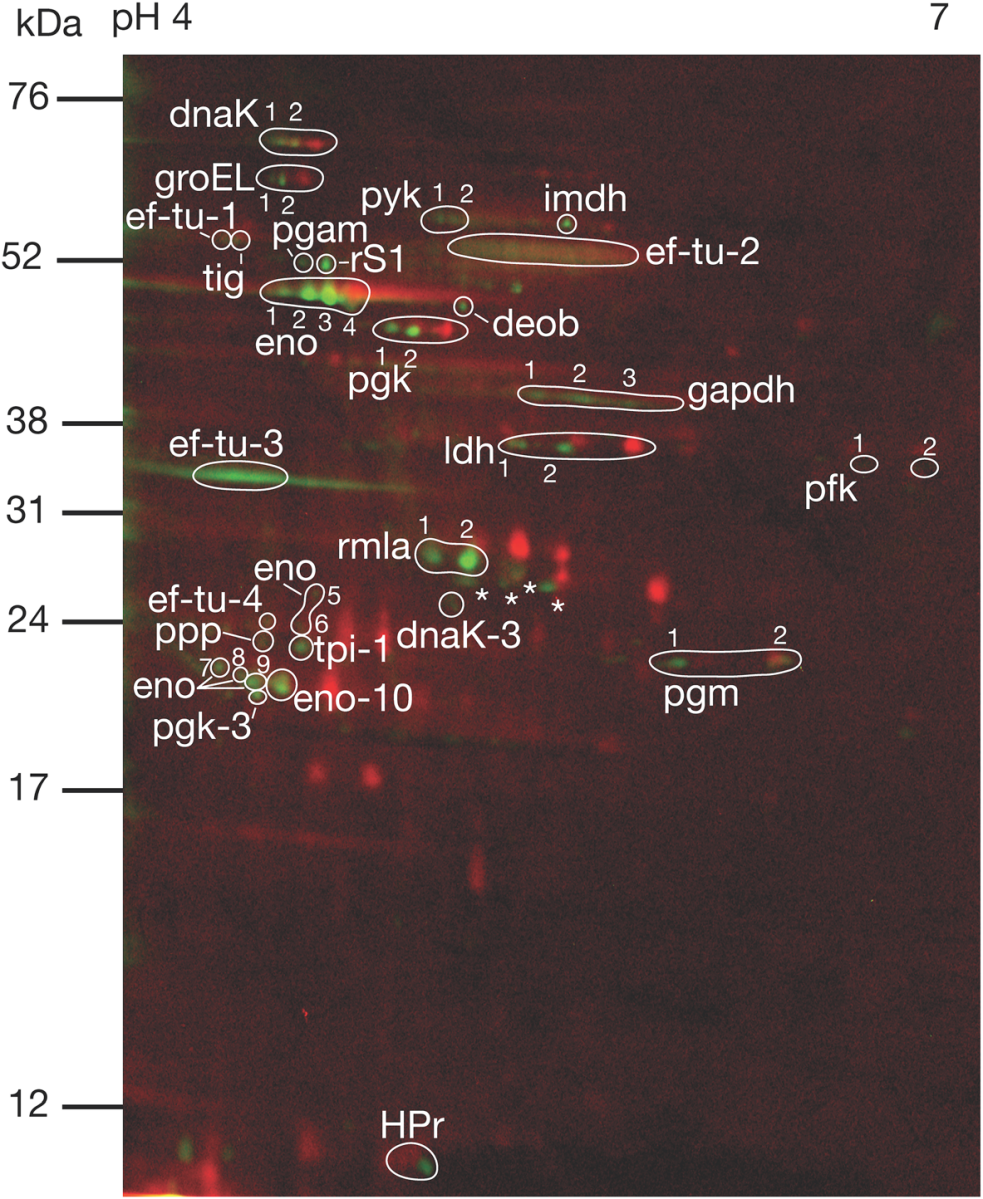
Representative 2DE phosphoprotein profile extracted from *S. gordonii *DL1. The red stain indicates intracellular proteins visualized with T-Rex Protein Labelling kit detecting lysine residues. The green stain indicates phosphorylated proteins visualized with Pro-Q Diamond Phosphoprotein Gel Stain detecting phosphate groups attached to serine, threonine or tyrosine residues by O-phosphorylation. Phosphorylated proteins identified by mass spectrometry are indicated (abbreviations are listed in Table 1). Phosphorylated spots with no significant hits in the Mascot database search are labelled with an asterisk (*). Gels were produced in triplicates from three different cultures of *S. gordonii* DL1

Spots containing Ser/Thr/Tyr phosphorylated proteins were visualized with Pro-Q Diamond stain and phosphoimaging. The total intracellular proteome was visualized with T-Rex protein labelling for orientation of the phosphorylated spots. In total, 49 phosphorylated spots were detected. These were generally found on the acidic side of the 2DE gels (Fig. 2).

Phosphorylated protein spots where identified with LC-MS/MS (Table 1). Six of the 49 phosphorylated spots remained unidentified, due to a lack of significant hits from the Mascot search (four spots) or absence from Coomassie gels, preventing excision for identification (two spots). In total, 19 putative Ser/Thr/Tyr phosphorylated proteins were identified, see Table 1.

### Cellular processes associated with Ser/Thr/Tyr phosphorylated proteins

**Fig. 3:**
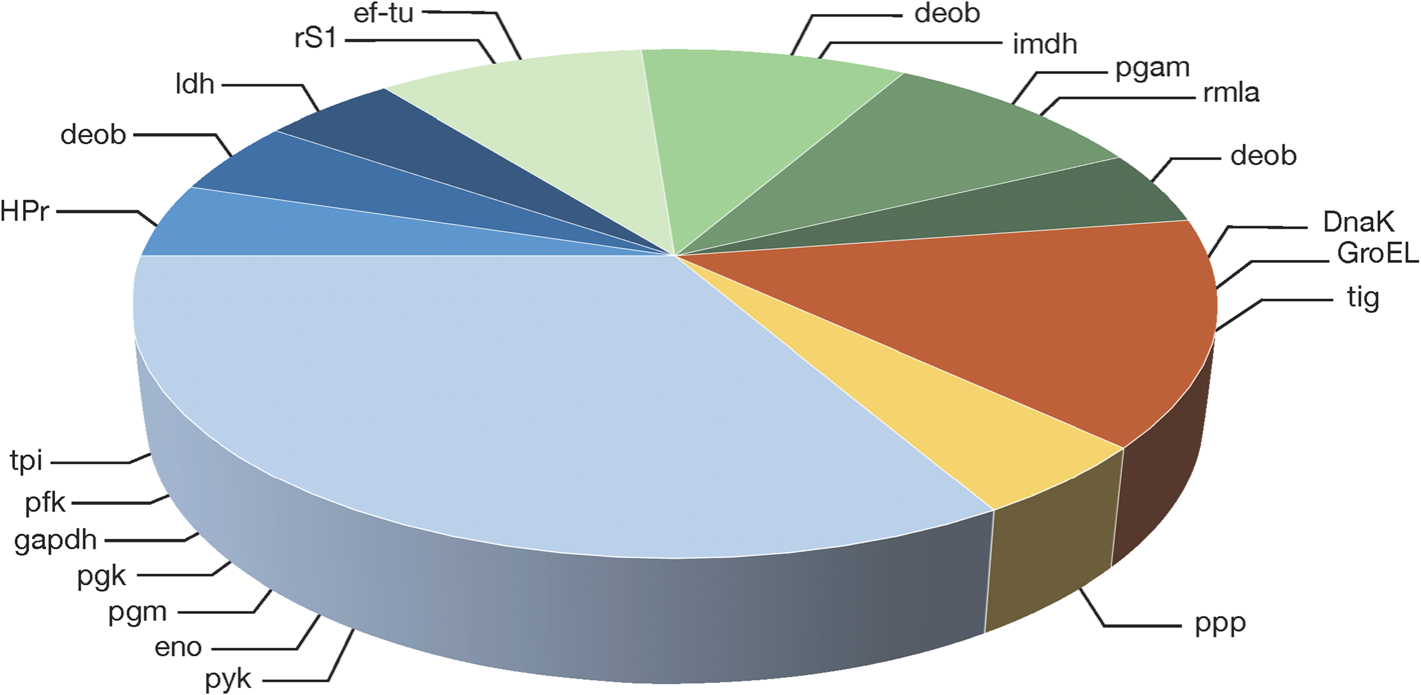
Ser/Thr/Tyr O-phosphorylated proteins involved in various cellular functions in *S. gordonii* DL1. Blue slices; carbon metabolism. Green slices; biosynthesis. Orange slice; chaperone function. Yellow slice; protein dephosphorylation.  glycolysis,  carbohydrate transport,  pentose phosphate pathway,  pyruvate conversion,  protein translation,  nucleotide biosynthesis,  cell wall biosynthesis,  amino acid biosynthesis,  molecular chaperone function,  protein dephosphorylation

The proteins detected as Ser/Thr/Tyr phosphorylated are involved in various cellular processes (Fig. 3). All phosphoproteins involved in the carbon metabolism, except for HPr and tpi, were present as more than one phosphorylated species, occurring at different p*I*s on the gels. HPr and tpi were also present as one additional, non-phosphorylated species respectively (Figs. 1 and 2, Table 1). Pfk and pgm were both present as two species each, all phosphorylated. Enolase was identified in both unphosphorylated and phosphorylated forms. Four of the phosphorylated enolase species were found at the expected MW. Pyk was present as four adjacent species, two of which were phosphorylated. Eight spots were identified as gapdh, three of which were phosphorylated. Nine species of pgk were identified, of which three were phosphorylated. Three species of ldh were identified, two phosphorylated. Six phosphorylated proteins involved in biosynthesis were identified. Deob, pgam and rS1 were all present as single phosphorylated species, and one of the two species of imdh was phosphorylated. Like enolase, ef-tu was found at several p*I*s, of which four were phosphorylated. Rmla was detected as two phosphorylated species. Three phosphorylated chaperones were identified. Six species of DnaK were identified, three of which were phosphorylated. GroEL was present as three species, two of which were phosphorylated. Tig was present as a single phosphorylated species. The protein phosphatase SGO_0599 (ppp) was detected as one phosphorylated species.

### Comparison of Ser/Thr/Tyr phosphoproteomes to other bacteria as detected with Pro-Q Diamond Phosphoprotein gel stain on 2DE-gels

To investigate whether the identified phosphoproteome of *S. gordonii* DL1 coincided with phosphoproteomes of other bacteria, literature searches were performed in the PubMed, Web of Science and Cochrane databases. Six studies were identified that examined the global Ser/Thr/Tyr phosphoproteomes in human commensal or pathogenic bacteria using 2DE gels and Pro-Q Diamond stain. Two studies on the model organism *Bacillus subtilis* were also found and included (Table 2). From planktonic cells grown under varying conditions, the previous studies revealed in total 27 and 29 putative Ser/Thr/Tyr phosphorylated proteins in *B. subtilis*, 15 in *L. rhamnosus*, 51 in *Neisseria meningitidis*, 73 in *Staphylococcus aureus*, and between 10 and 26 in different species of mycoplasma. All phosphoproteins identified in the current study except for three (pfk, rmla, ppp), were also detected as Ser/Thr/Tyr-phosphorylated in those studies, with varying occurrences. The number of phosphorylated proteins in *S. gordonii* DL1 detected in this study make up approximately 1% of the total number of annotated proteins in the UniProt database [45] for this species. This corresponds well to the other species tested with similar methodology, whose Ser/Thr/Tyr-phosphoproteomes as detected with Pro-Q Diamond stain varied between 0.7 to 2.6% of the total proteomes. Confirmed or putative Ser/Thr/Tyr phosphorylation sites in all proteins identified as phosphorylated in this study or homologues, except for rmla and ppp, were found in the literature (Table 2).

**Table 2:** Comparison to Ser/Thr/Tyr phosphoproteomes of other bacteria as detected with Pro-Q Diamond Phosphoprotein Gel Stain on 2DE gels

Protein	Protein also detected as phosphorylated with Pro-Q Diamond in	Number of phosphorylated protein species
**Carbohydrate transport**		
HPr^b^ [7, 25, 26]	*B. subtilis* 168 trpC2 [27] *M. penetrans* GTU-54 [28]	1 1
**Glycolysis**		
tpi^b^ [29, 30]	*L. rhamnosus* GG (ATCC 53103) [19] *M. penetrans* GTU-54 [28]	1 1
pfk^b^ [31]	–	–
gapdh^b^ [29, 30]	*B. subtilis* 168 trpC2 [27] *L. rhamnosus* GG (ATCC 53103) [19] *S. aureus* COL [32] *N. meningitidis* Z4970 (serogroup A) [33] *M. penetrans* GTU-54 [28]	1 2 3 2 1
pgk^b^ [29, 34, 35]	*B. subtilis* 168 trpC2 [27] *L. rhamnosus* GG (ATCC 53103) [19] *N. meningitidis* Z4970 (serogroup A) [33] *S. aureus* COL [32]	1 1 2 2
pgm^b^ [29, 34]	*B. subtilis* 168 trpC2 [27] *S. aureus* COL [32] *M. penetrans* GTU-54 [28]	2 3 1
eno^b^ [20, 29, 30, 34, 36]		
	*B. subtilis* 168 trpC2 [27] *B. subtilis* 168 trpC2 [37] *S. aureus* COL [32] *N. meningitidis* Z4970 (serogroup A) [33]	3 3 3 2
pyk^b^ [29, 30, 34, 38, 39]	*B. subtilis* 168 trpC2 [27] *B. subtilis* 168 trpC2 [37] *L. rhamnosus* GG (ATCC 53103) [19] *S. aureus* COL [32] *M. penetrans* GTU-54 [28]	4 1 1 5 1
**Pyruvate conversion**		
ldh^a^ [29, 35]	*M. genitalium* G37 (ATCC 33530) [40] *M. pneumoniae* M129-B170 (ATCC 29343) [40]	2 1
**Pentose phosphate pathway, amino acid and nucleotide biosynthesis**		
deob^b^ [34, 35]	*S. aureus* COL [32] *B. subtilis* 168 trpC2 [27]	2 1
**Nucleotide biosynthesis**		
imdh^b^ [41]	*B. subtilis* 168 trpC2 [27]	2
**Molecular chaperone activity**		
DnaK^a^ [30, 34]	*B. subtilis* 168 trpC2 [27] *N. meningitidis* Z4970 (serogroup A) [33] *M. penetrans* GTU-54 [28] *M. gallisepticum* S6 [42] *M. genitalium* G37 (ATCC 33530) [40] *M. pneumoniae* M129-B170 (ATCC 29343) [40]	1 2 1 1 1 1
GroEL^b^ [30, 35, 39]	*B. subtilis* 168 trpC2 [27] *L. rhamnosus* GG (ATCC 53103) [19] *N. meningitidis* Z4970 (serogroup A) [33] *M. penetrans* GTU-54 [28] *M. gallisepticum* S6 [42] *M. genitalium* G37 (ATCC 33530) [40] *M. pneumoniae* M129-B170 (ATCC 29343) [40]	1 1 4 1 1 1 1
Tig^b^ [19]	*L. rhamnosus* GG (ATCC 53103) [19] *B. subtilis* 168 trpC2 [27] *B. subtilis* 168 trpC2 [37] *S. aureus* COL [32] *N. meningitidis* Z4970 (serogroup A) [33] *M. pneumoniae* M129-B170 (ATCC 29343) [40]	1 1 1 1 1 1
**Protein translation**		
ef-tu^b^ [30, 43]	*B. subtilis* 168 trpC2 [27] *B. subtilis* 168 trpC2 [37] *S. aureus* COL [32] *N. meningitidis* Z4970 (serogroup A) [33] *M. penetrans* GTU-54 [28] *M. genitalium* G37 (ATCC 33530) [40] *M. pneumoniae* M129-B170 (ATCC 29343) [40]	1 1 1 2 1 1 1
rS1^b^ [19]	*L. rhamnosus* GG (ATCC 53103) [19] *N. meningitidis* Z4970 (serogroup A) [33]	1 2
**Cell envelope biosynthesis**		
pgam^a^ [44]	*S. aureus* COL [32]	2
rmla	–	–
**Protein dephosphorylation**		
ppp	–	–

^a^Phosphorylation site on Ser/Thr/Tyr identified in *S. gordonii* DL1 according to the UniProt.org database [45]

^b^Ser/Thr/Tyr phosphorylation site suggested in other species, references are listed in parenthesis

Of the 19 identified putative phosphoproteins in *S. gordonii* DL1, 13 were replicated in *B. subtilis*, 10 in *S. aureus*, 8 in *N. meningitidis*, 7 in *L. rhamnosus*, 8 in *M. penetrans*, 5 in *M. pneumoniae*, 4 in *M. genitalium* and 2 in *M. gallisepticum*. As in *S. gordonii* DL1, several of the phosphorylated proteins were also present at two or more p*I*s in these bacteria (Table 2).

## Discussion

### Ser/Thr/Tyr phosphoproteome of *S. gordonii* DL1

Putative Ser/Thr/Tyr phosphorylated intracellular proteins in *S. gordonii* DL1 were identified from 2DE gels using Pro-Q Diamond stain and LC-MS/MS. From the 49 phosphorylated spots detected, 19 Ser/Thr/Tyr phosphoproteins were identified in *S. gordonii*. Determined or putative phosphorylation sites on Ser, Thr and/or Tyr in all phosphorylated proteins or homologues except for two, rmla and ppp, were found in the literature (Table 2).

A variety of cellular processes were associated with the phosphoproteins identified in *S. gordonii* DL1 (Fig. 3). This supports the notion that Ser/Thr/Tyr phosphorylation events are integrated in the pathways that regulate different cellular responses in this species. Many of the phosphorylated proteins were present at two or more p*I*s in *S. gordonii*. As is evident from Table 2, this is a common occurrence in other bacteria as well. Variably phosphorylated species of proteins are of interest because they may differ in function [21] and hence be of biological relevance. With 2DE, although laborious, the occurrence of physiologically distinct forms of one protein can be readily visualized in a more “hands on”, comprehensible overview of the present proteomic profile, were differently modified protein species often occur at adjacent p*I*s. Studies on deletion mutants can be employed to investigate the role of specific kinases/phosphatases on phenotypic presentation or different bacterial functions [46]. The current material constitutes a first step towards future studies that may utilize such targeted approaches. Studies of this character can be carried out from any perspective of microbial physiology and biological interaction of interest. Future studies using clinical isolates as well as biofilm growth flow cell models with saliva as a nutritious substrate are also of interest to increase the resemblance to in vivo conditions.

### Core phosphoproteome

The total number of identified Ser/Thr/Tyr phosphorylated proteins as detected with the Pro-Q Diamond stain in other bacteria grown in various growth conditions was between 10 and 73 [19, 27, 28, 32, 33, 37, 40, 42], making up between 0.7 to 2.6% of the total proteomes as annotated in the UniProt database [45]. This is in accordance with the 19 phosphoproteins identified in *S. gordonii* DL1 (approximately 1% of the total proteome). All phosphoproteins identified in the current study except for three (pfk, rmla, ppp) were also previously detected as Ser/Thr/Tyr-phosphorylated with the Pro-Q Diamond stain in other bacteria (Table 2). Proteins that are consistently found in their phosphorylated state across several species and growth conditions may represent a core phosphoproteome profile shared by many bacteria. The mapping of such a core phosphoproteome may facilitate the identification of phenotypic characteristics that deviate from this core pattern. Identification of distinct phenotype phosphorylation patterns that reflect microbial activities may be crucial for pursuing a further understanding of biofilm formation and colonization of the commensal microbiota. Based on our findings, pfk, rmla and ppp represent possible proteins of interest for further investigation. Two phosphorylated protein species of pfk were detected in *S. gordonii*. In *Streptococcus pneumoniae*, pfk was found to be phosphorylated on a tyrosine residue, and phosphorylation of this enzyme was suggested to be involved in regulation of the metabolic flux in the cell [31]. One phosphorylated protein species of ppp was detected in *S. gordonii*. The phosphatase stp1 in *Streptococcus agalactiae* was identified as a homologue to *S. gordonii* ppp (100% similarity) in the UniProt.org database [45]. Stp1 is involved in group B streptococcal virulence [47]. Two phosphorylated protein species of rmla was identified in *S. gordonii*. Activation and deactivation of rmla is an important regulating mechanism of peptidoglycan biosynthesis in gram positive bacteria, making it an interesting candidate in the search for new antibiotics [48]. Hardly anything can be found in the literature about potential regulation of catalytic activity by phosphorylation of rmla, but the current findings suggest that Ser/Thr/Tyr phosphorylation events are involved. These three proteins represent possible candidates for uniquely phosphorylated proteins in *S. gordonii,* however, because phosphorylation patterns are dependent on growth conditions, further experiments comparing the phosphoproteomes of different species grown in the same environments are needed.

### Phosphoproteins in *S. gordonii* DL1 involved in carbohydrate transport and metabolism

The carbohydrate transporting protein HPr was detected as Ser/Thr/Tyr phosphorylated in *S. gordonii*. HPr is a known phosphoprotein with roles in the phosphotransferase system (PTS), for uptake and phosphorylation of certain carbohydrates in the central carbon metabolism. The PTS has a high affinity for glucose, and a preference for glucose over alternative carbohydrates. HPr carries known phosphorylation sites on histidine, *per se* involved in the phosphotransfer catalytic activity, and serine, involved in the *regulation* of catalytic phosphotransfer activity [49]. Phosphorylation on HPr serine reduces the phosphotransfer activity, thereby reducing the uptake of PTS-carbohydrates, while simultaneously enhancing the uptake of alternative carbohydrates [7, 26, 49]. In this way, phosphorylation events on HPr can regulate both the quantity and types of carbohydrates that are processed by the cell. Seven glycolytic proteins (tpi, pfk, gapdh, pgk, pgm, eno, pyk), as well as ldh, involved in pyruvate conversion, were Ser/Thr/Tyr phosphorylated in *S. gordonii*.

Prokaryotes have been suggested to regulate glycolytic activity rapidly and reversibly through protein phosphorylation and dephosphorylation in response to environmental changes [12, 13]. Our findings indicate that these mechanisms are present in *S. gordonii* as well. Aside from controlling the metabolic rate and character of the metabolic end products of glycolysis, *e.g.* by activation of ldh, regulation of some glycolytic enzymes, *e.g.* tpi, controls the switch between different metabolic pathways [50, 51]. Regulation of enzymes with reversible catalytic function, *e.g.* pgk, eno, may enable switching between catabolism and anabolism [45]. Rapid and reversible regulation of carbohydrate transport, control of rate of glycolysis, and alternative carbohydrate metabolizing pathways by phosphorylation, increase cell fitness by enabling adaptation of the cell metabolism in response to variations in carbohydrate concentration. Thereby, damage from processes such as sugar killing can be mitigated [8, 9, 12]. Our findings indicate that Ser/Thr/Tyr phosphorylation is involved in regulating the activities of these proteins. Control of glycolysis not only by the levels of metabolic substrates and products, but by transduction of a variety of other signals through enzyme phosphorylation events, suggests a high complexity of metabolic control in *S. gordonii*. This supports the idea that integration of metabolic activity and environmental factors in bacterial cells is more convoluted than previously believed.

### Phosphoproteins in *S. gordonii *DL1 involved in acid tolerance response

To cope with the intermittent fluctuations in pH that naturally occur in oral biofilms, partly as a consequence of accumulation of lactic acid and other acids from the central carbon metabolism, oral streptococci employ the so-called acid tolerance response (ATR) [4, 10, 11]. Aside from responsive regulation of carbohydrate transport and metabolism, increased molecular chaperone activity comprises another important aspect of the ATR in oral streptococci [4, 8]. The chaperones represent the group of proteins most frequently detected as Ser/Thr/Tyr phosphorylated in other species (Table 2). In the current study, *S. gordonii* displayed three phosphorylated molecular chaperones (DnaK, GroEL, tig). Ser/Thr/Tyr phosphorylation has been suggested to activate all these three chaperones in bacteria [27, 37, 39, 52]. By modulating the character and acidity of metabolic end products that accumulate locally, while retaining effective metabolism and other cellular functions at lower environmental pH with the help of ATR responses, cells increase their competitiveness towards less aciduric species [53]. From an evolutionary point of view, these mechanisms may have contributed to the long-term survival of these species in the strenuous oral environment.

### Phosphoproteins in *S. gordonii *DL1 involved in biosynthesis

Six phosphorylated proteins involved in biosynthesis were identified in *S. gordonii* DL1 (deob, imdh, ef-tu, rS1, pgam, rmla). These proteins are involved in amino acid, nucleotide and cell wall biosynthesis, as well as protein translation (Table 1). Except for the chaperones, ef-tu was the protein most commonly detected with Pro-Q Diamond stain on 2DE gels in the previously studied bacteria (Table 2). Phosphorylation on a threonine residue in *Escherichia coli* ef-tu seems to inactivate the protein by decoupling the tRNA from the ribosome A-site [54, 55], triggering a rapid inhibition of protein translation. In contrast, phosphorylation on a threonine residue in the ribosomal protein rS1 in *E. coli* was found to activate protein translation [56]. Rapid and reversible regulation of biosynthesis through protein phosphorylation events enables the cell to be frugal regarding energy expenditure, and streamline cellular functions by modification of biomolecule production.

### Moonlighting and multisite regulation of identified phosphoproteins

Several of the proteins identified as phosphorylated in *S. gordonii* DL1 have known moonlighting functions, often in adhesion, *e.g.* tpi [57], gapdh [58], eno [59], and GroEL [60], alternative metabolic pathways, *e.g.* gapdh in iron metabolism [61], or other functions. In prokaryotes, cell surface associated enolase is involved in adhesion to host components in connective tissue or saliva [59], and retention of exported enolase on the bacterial cell surface was found to increase in acidic environments [58]. Maintaining cell adhesion during acidic shifts that may inactivate other adhesins is a tentative strategy for increased competitiveness in oral biofilms. In *E. coli* enolase, apart from reducing its glycolytic activity, lysine phosphorylation also prevented the enzyme export related to its moonlighting functions [62]. These findings suggest that phosphorylation of enolase may affect cell adhesion during acidic shifts. Regulation of several of the phosphoproteins identified in *S. gordonii* DL1, such as chaperones DnaK and GroEL, also seem to involve multisite phosphorylation events [30, 34, 39, 63]. This further supports the idea that regulation of cellular activities in prokaryotes is complex and involves integrated patterns of signal transduction.

## Conclusion

This study clearly shows that Ser/Thr/Tyr phosphorylation is present in an array of cytoplasmic proteins from the oral commensal *S. gordonii*. In total, 61 intracellular proteins were identified in *S. gordonii* DL1, and 19 of these turned out to be present as phosphorylated. Most of the phosphorylated proteins are involved in the carbon metabolism, specifically related to glycolysis, which is no surprise for this saccharolytic oral streptococcus. Ser/Thr/Tyr phosphorylation presents a possible mechanism for regulation of multiple cellular processes in *S. gordonii*, and phosphorylated proteins species were often present at several p*I*s with potential variation in biological function. Many similarities were found between the identified phosphoproteome of *S. gordonii* DL1 and that of previously studied species, despite differences in basic cell physiology and the growth conditions applied. Identification of core regulatory pathways involved in the interaction between bacteria and their environment should provide useful insights regarding new strategies to manage biofilm-induced diseases. Studies in microbiology are often focused on investigating the role of bacteria in disease. However, the physiology of commensals that prevent shifts towards dysbiosis are just as relevant. To investigate mechanisms of biofilm formation and homeostasis in the oral cavity, it is essential to examine bacterial responses and regulation of responses in relation to specific environmental challenges.

## Methods

### Bacterial strain and culture conditions

*S. gordonii* DL1 was routinely grown overnight in 25% Todd-Hewitt Yeast Extract (¼ THYE, Becton Dickinson), at 37 °C in 5% CO_2_. Cell cultures were diluted 1:10 in 25% THYE + 20 mM glucose (¼ THYE+G) and grown as described above until the mid-exponential phase (OD600nm = 0.5–0.6) was reached. Planktonic cells were retrieved by centrifugation (3000 rpm, 10 min, 5 °C), washed, and resuspended in a 10 mM Tris HCl-buffer pH 6.8 containing 1 mM EDTA and 5 mM MgSO_4_, and stored at − 20 °C until protein extraction.

### Protein extraction

Harvested cells were subjected to three freeze-thaw cycles before washing and then resuspended in 700 μl lysis buffer containing 8 M urea, 2% (v/v) CHAPS, 64.8 mM DTT, 2% IPG buffer pH 4–7 (Pharmacia Amersham Biotech, Sweden). Ultrasonication of the samples was then performed with homogenizing 0.2 mm glass beads for 4 × 5 min (5 s pulses, amplitude 40, Vibra-Cell™ Ultrasonic Processor, SONICS), with alternate periods of cooling. Intact cells were sedimented by centrifugation at 17000×g for 10 min at 4 °C and the supernatants (protein extracts) were stored at − 20 °C. The protein concentration was determined using the 2-D Quant kit (GE Healthcare Life Sciences).

### 2D gel electrophoresis

Intracellular proteins were extracted and separated by two-dimensional sodium dodecyl sulfate-polyacrylamide gel electrophoresis (2D SDS-PAGE; 2DE) as described previously [64]. Isoelectric focusing was carried out on 18 cm Immobiline Dry Strips pH 4–7 (Amersham Pharmacia Biotech, Sweden) followed by gel electrophoresis on 14% polyacrylamide gels (185 × 200 × 1.0 mm). Gels for Coomassie staining were loaded with 150 μg protein. Gels for fluorescent staining were loaded with 50 μg protein, and proteins were pre-labelled with the T-Rex Labelling kit (NH DyeAGNOSTICS) before rehydration (see “Staining procedures” below). The gels were then fixed in the appropriate fix solution as recommended by the respective stain manufacturer (Coomassie stain 2% acetic acid and 40% ethanol and fluorescent stain 10% acetic acid and 50% ethanol). Gels for Coomassie staining were fixed for at least 1 h and gels for fluorescent staining were fixed overnight, protected from light. All gels were produced in triplicates from separate cell cultures.

### Staining procedures

Coomassie gels were stained overnight in 300 ml Coomassie brilliant blue staining solution containing 17% Coomassie Brilliant Blue G - Colloidal Concentrate (Sigma) and 21% ethanol. After staining, Coomassie gels were destained with 25% ethanol for approximately 1 h and stored in Ultra-High Quality water (UHQ) at 4 °C until scanning and excision of spots. For fluorescent staining, lysine residues of the general proteome were pre-labelled with T-Rex Labelling kit (NH DyeAGNOSTICS) before rehydration, to facilitate orientation of the phosphorylation profiles. Pre-labelling was performed according to the manufacturer’s protocol. Briefly, 2 μl T-Rex solvent and 50 μg of the extracted proteins were transferred into a T-Rex labelling vial and incubated on ice for 30 min. Thereafter the gels were prepared as described above. After fixation, each T-rex stained gel was washed with UHQ for 3 × 15 min and pre-scanned once at 647 nm with a photomultiplier tube (PMT) setting of 800, pixel size 100 μm, before staining with Pro-Q Diamond Phosphoprotein Gel Stain (Invitrogen™, ThermoFisher Scientific) according to the manufacturer’s instruction. Phosphoprotein staining was performed protected from light, and with minor modifications based on results from Agrawal and Thelen [65]. Each gel was stained with Pro-Q Diamond Phosphoprotein Gel Stain diluted 1:2 in UHQ to a final volume of 250 ml for 2 h. Gels were destained in 250 ml Pro-Q Diamond Phosphoprotein Gel Destaining Solution (Invitrogen™, ThermoFisher Scientific) per gel for 3 × 30 min on a shaker and then washed in UHQ for 3 × 5 min.

### Phosphoimage analysis

Phosphoimaging was performed with a Fujifilm FLA-9000 (Science Imaging Scandinavia AB). T-Rex labelled proteins were visualized at 647 nm, PMT 800, 100 μm (displayed as red). Pro-Q Diamond stained proteins were visualized at 532 nm, PMT 600, 100 μm (displayed as green). Phosphorylation profiles were analysed by three operators and then compared and discussed for calibration, with good concordance. Quantification of Pro-Q Diamond Phosphoprotein Gel Stain signals from phosphorylated spots was not possible due to variations between triplicates.

### Identification of streptococcal proteins by LC-MS/MS

Protein spots were manually excised from Coomassie brilliant blue-stained gels and subjected to in-gel digestion with trypsin as previously described [64]. The resulting protein fragments were separated with liquid chromatography (LC) and characterized using tandem mass spectrometry (MS/MS) (Aberdeen Proteomics, University of Aberdeen). In short, proteins were treated with DTT for reduction (60 °C, 20 min), iodacetamide for S-alkylation (25 °C, 10 min) and trypsin for digestion (37 °C, 8 h). Following drying by rotary evaporation (SC110 Speedvac, Savant Instruments), the peptide extract was dissolved in 0.1% formic acid. Analysis of peptide solutions was carried out using an HTCultraPTM Discovery system (Bruker Daltonics) coupled to an UltiMate 3000 LC system (Dionex). Separation of tryptic peptides was performed on a monolithic capillary column (200 μm internal diameter × 5 cm, Dionex). The gradient consisted of 5% acetonitrile in UHQ containing 0.5% formic acid, to 37% acetonitrile in UHQ containing 0.45% formic acid over 12 min at a flow rate of 2.5 μl min^− 1^. Data-dependent mode was employed to acquire peptide fragment mass spectra, AutoMS (2). The scan range was 300–1500 m/z, with three averages, and up to three precursor ions that were selected from the MS scan (100–2200 m/z). Active exclusion of precursors was performed within a 1.0 min window, as well as exclusion of all singly charged ions. Detection and deconvolution of peptide peaks were carried out automatically using Data Analysis software (Bruker). Mass lists in the form of Mascot Generic files were created automatically and used as the input for Mascot MS/MS ion searches in the NCBInr database for Firmicutes (Gram Positive Bacteria) using the Matrix Science web server [66]. Parameters were set to 0.5 Da peptide mass tolerance, methionine oxidation and carboxyamidomethylation of cysteine. The maximum number of missed cleavages was set to 1. Proteins were identified by at least two peptides with a Mascot score of 33 or higher, the latter as suggested by Koenig et al., 2008 [67].

### Literature search

The PubMed, Web of Science and Cochrane databases were searched for studies that examined global Ser/Thr/Tyr phosphoproteomes in human commensal and pathogenic bacteria using Pro-Q Diamond Phosphoprotein Gel Stain on 2DE gels. Studies on the model organism *Bacillus subtilis* were also included.

## Supplementary information

**Additional file 1.** The sequenced peptides identified by LC-MS/MS can be found in supplemental material (Additional file 1). Sequenced peptides identified by LC-MS/MS; Ser/Thr/Tyr phosphorylated protein species (Table A), and non-phosphorylated protein species (Table B).

## Data Availability

All datasets generated or analysed during this study are included in this published article and its supplementary information files, see (Additional file 1). The gel triplicates are available from the corresponding author on reasonable request.

## Abbreviations

2DE: Two-dimensional gel electrophoresis
¼ THYE: 25% Todd-Hewitt Yeast Extract
¼ THYE+G: 25% THYE + 20 mM glucose
ATR: Acid tolerance response
MW: Molecular weight
PTS: Phosphotransferase transport system
Ser/Thr/Tyr: Serine, threonine, tyrosine
UHQ: Ultra-High Quality water
