# Supplementary material for: Intracellular Ser/Thr/Tyr phosphoproteome of the oral commensal Streptococcus gordonii DL1
Source: BMC Microbiol. 2020 Sep 14;20:280. doi: 10.1186/s12866-020-01944-y (PMC7488673; doi:10.1186/s12866-020-01944-y)
Supplement: Supplementary file 1 — Additional file 1. The sequenced peptides identified by LC-MS/MS can be found in supplemental material (Additional file 1). Sequenced peptides identified by LC-MS/MS; Ser/Thr/Tyr phosphorylated protein species (Table A), and non-phosphorylated protein species (Table B). [file 12866_2020_1944_MOESM1_ESM.docx]

**Supplemental material**

The sequenced peptides identified by LC-MS/MS for protein identification are listed below in alphabetic order. Table A lists Ser/Thr/Tyr phosphorylated protein species and Table B non-phosphorylated protein species.

| **Table A. Ser/Thr/Tyr phosphorylated protein species.** | |
| --- | --- |
| Deob | DCLEEFDAR, LNEAGIDTYSVGK, EANKPYSGTAVIDDFGPR. |
| DnaK-1 | GYAEEYLGEK, FQLTDIPAAPR, IVNEPTAAALAYGLDK, EQTIVIQSNSGLTDEEIDR. |
| DnaK-2 | IAGLEVER, IPAVVEAVK, AKFDDLTR, IIANPEGNR, LGGDDFDQK, ENGIDLSTDK, GYAEEYLGEK, FQLTDIPAAPR  NGEIIVGDAAKR, DAGKIAGLEVER, LIDRNTTIPTSK, LEALNEKAQGLAVK, GYAEEYLGEKVTK, RQAVTNPDTVISIK, AVITVPAYFNDAQR, IVNEPTAAALAYGLDK, ENGIDLSTDKMoxALQR, EYTPQEISAMILQYLK, EQTIVIQSNSGLTDEEIDR, GIPQIEVTFDIDKNGIVSVK, IIGIDLGTTNSAVAVLEGTESK, SVNPDEVVAMGAAIQGGVITGDVK, SKIIGIDLGTTNSAVAVLEGTESK, QALSDAGLSLSEIDEVILVGGSTR, VSANGKEYTPQEISAMoxILQYLK, DLGTQKEQTIVIQSNSGLTDEEIDR. |
| DnaK-3 | IAGLEVER, IVNEPTAAALAYGLDK. |
| Ef-tu-1 | AVVTGVEMFR, VNDEIEIVGIK, TTLTAAITTVLAR, DYASIDAAPEER, QLDEGLAGDNVGVLLR, DTDKPLLLPVEDVFSITGR, VDLVDDEELLELVEMoxEIR, NMoxITGAAQMoxDGAILVVASTDGPMoxPQTR. |
| Ef-tu-2 | EHILLSR, HLIVFMNK, AVVTGVEMFR, RLPSSVNQPK, FKGEVYILTK, VNDEIEIVGIK, TTLTAAITTVLAR, DYASIDAAPEER, GQVIAKPGSINPHTK, DYASIDAAPEERER, QLDEGLAGDNVGVLLR, HYAHIDAPGHADYVK, SKPHVNIGTIGHVDHGK, KQLDEGLAGDNVGVLLR, VNDEIEIVGIKEEIQK, GITINTAHVEYETEKR, DTDKPLLLPVEDVFSITGR, VDLVDDEELLELVEMoxEIR, DLLSEYDFPGDDLPVIQGSALK, NMITGAAQMoxDGAILVVASTDGPMPQTR, HLIVFMoxNKVDLVDDEELLELVEMoxEIR. |
| Ef-tu-3 | AVVTGVEMFR, VNDEIEIVGIK, TTLTAAITTVLAR, QLDEGLAGDNVGVLLR, DLLSEYDFPGDDLPVIQGSALK, YEDIIMoxDLMoxNTVDEYIPEPER. |
| Ef-tu-4 | AVVTGVEMFR, VNDEIEIVGIK, DYASIDAAPEER. |
| Eno-1 | FEGEGAAVR, SIITDVYAR, GLQAFYNLK, AMIALDGTPNK, YGAEIFHALK, EAGYTAVVSHR, IEDQLGEVAQYK, LGANAILGVSIAVAR, GLETAVGDEGGFAPR, TAAEQIDYLEELVNK, GMVPSGASTGEHEAVELR, DVFIGFDCASSEFYDK, GNPTLEVEVYTESGAFGR, VNQIGTLTETFDAIEMAK, AVDNVNNVIAEAIIGYDVR, VQLVGDDFFVTNTDYLSR, SGETEDSTIADIAVATNAGQIK, AAADYLEIPLYSYLGGFNTK. |
| Eno-2 | IEDQLGEVAQYK, LGANAILGVSIAVAR, GLETAVGDEGGFAPR, TAAEQIDYLEELVNK, GMoxVPSGASTGEHEAVELR, DVFIGFDCASSEFYDK, GNPTLEVEVYTESGAFGR, VNQIGTLTETFDAIEMAK, AVDNVNNVIAEAIIGYDVR, VQLVGDDFFVTNTDYLSR, SGETEDSTIADIAVATNAGQIK, AAADYLEIPLYSYLGGFNTK, FEGTEDGVETIIAAIEAAGYVPGK. |
| Eno-3 | AMoxIALDGTPNK, EAGYTAVVSHR, IEDQLGEVAQYK, LGANAILGVSIAVAR, GLETAVGDEGGFAPR, GKLGANAILGVSIAVAR, TAAEQIDYLEELVNK, GMVPSGASTGEHEAVELR, DVFIGFDCASSEFYDK, GNPTLEVEVYTESGAFGR, VNQIGTLTETFDAIEMoxAK, AVDNVNNVIAEAIIGYDVR, VQLVGDDFFVTNTDYLSR, SGETEDSTIADIAVATNAGQIK, AAADYLEIPLYSYLGGFNTK, KVQLVGDDFFVTNTDYLSR, YPIITIEDGMDENDWDGWK, FEGTEDGVETIIAAIEAAGYVPGK. |
| Eno-4 | YNQLLR, YGGLGTQK, FEGEGAAVR, SIITDVYAR, GLQAFYNLK, EGCANSILIK, AMoxIALDGTPNK, YGAEIFHALK, GLQAFYNLKK, EAGYTAVVSHR, YGAEIFHALKK, AMoxIALDGTPNKGK, IEDQLGEVAQYK, GIKEGCANSILIK, LGANAILGVSIAVAR, GLETAVGDEGGFAPR, GKLGANAILGVSIAVAR, SRGLETAVGDEGGFAPR, TAAEQIDYLEELVNK, GMoxVPSGASTGEHEAVELR, GNPTLEVEVYTESGAFGR, VNQIGTLTETFDAIEMAK, AVDNVNNVIAEAIIGYDVR, VQLVGDDFFVTNTDYLSR, DVFIGFDCASSEFYDKER, SGETEDSTIADIAVATNAGQIK, AAADYLEIPLYSYLGGFNTK, KVQLVGDDFFVTNTDYLSR, GMoxVPSGASTGEHEAVELRDGDK, FEGTEDGVETIIAAIEAAGYVPGK, AVDNVNNVIAEAIIGYDVRDQQAIDR. |
| Eno-5 | FEGEGAAVR, SIITDVYAR, GLQAFYNLK, AMIALDGTPNK, YGAEIFHALK, EAGYTAVVSHR, IEDQLGEVAQYK, LGANAILGVSIAVAR, GLETAVGDEGGFAPR, TAAEQIDYLEELVNK, DVFIGFDCASSEFYDK, GNPTLEVEVYTESGAFGR, VNQIGTLTETFDAIEMAK, VQLVGDDFFVTNTDYLSR, SGETEDSTIADIAVATNAGQIK, AAADYLEIPLYSYLGGFNTK, FEGTEDGVETIIAAIEAAGYVPGK. |
| Eno-6 | GLQAFYNLK, EGCANSILIK, EAGYTAVVSHR, IEDQLGEVAQYK, DVFIGFDCASSEFYDK, VNQIGTLTETFDAIEMAK, VQLVGDDFFVTNTDYLSR, SGETEDSTIADIAVATNAGQIK. |
| Eno-7 | GLQAFYNLK, EGCANSILIK, EAGYTAVVSHR, IEDQLGEVAQYK, TAAEQIDYLEELVNK, DVFIGFDCASSEFYDK, VNQIGTLTETFDAIEMoxAK, VQLVGDDFFVTNTDYLSR, SGETEDSTIADIAVATNAGQIK. |
| Eno-8 | FEGEGAAVR, GLQAFYNLK, EGCANSILIK, EAGYTAVVSHR, IEDQLGEVAQYK, TAAEQIDYLEELVNK, VNQIGTLTETFDAIEMoxAK, VQLVGDDFFVTNTDYLSR, SGETEDSTIADIAVATNAGQIK. |
| Eno-9 | FEGEGAAVR, GLQAFYNLK, EGCANSILIK, EAGYTAVVSHR, IEDQLGEVAQYK, TAAEQIDYLEELVNK, VNQIGTLTETFDAIEMoxAK, VQLVGDDFFVTNTDYLSR, SGETEDSTIADIAVATNAGQIK, YPIITIEDGMoxDENDWDGWK. |
| Eno-10 | FEGEGAAVR, GLQAFYNLK, EAGYTAVVSHR, IEDQLGEVAQYK, TAAEQIDYLEELVNK, DVFIGFDCASSEFYDK, VNQIGTLTETFDAIEMAK, VQLVGDDFFVTNTDYLSR, DVFIGFDCASSEFYDKER, SGETEDSTIADIAVATNAGQIK, YPIITIEDGMoxDENDWDGWK. |
| Gapdh-1 | VLDVDGK, VGINGFGR, TLEYFAK, FDGTVEVK, EGGFEVNGK, VVITAPGGSDVK, VLDVDGKQLVK, AIGLVIPELNGK, IQNVEGVEVTR, KVVITAPGGSDVK, NVTVDEVNAAMK, AGAANIVPNSTGAAK, RIQNVEGVEVTR, ARAGAANIVPNSTGAAK, INDLTDPVMLAHLLK, YDTTQGRFDGTVEVK, VPTPTGSVTELVVVLEK, FDGTVEVKEGGFEVNGK, AIGLVIPELNGKLDGAAQR, VVSWYDNEMoxSYTAQLVR. |
| Gapdh-2 | VGINGFGR, TLEYFAK, FDGTVEVK, EGGFEVNGK, VLDVDGKQLVK, AIGLVIPELNGK, IQNVEGVEVTR, KVVITAPGGSDVK, NVTVDEVNAAMK, AGAANIVPNSTGAAK, RIQNVEGVEVTR, ARAGAANIVPNSTGAAK, INDLTDPVMLAHLLK, YDTTQGRFDGTVEVK, VPTPTGSVTELVVVLEK, FDGTVEVKEGGFEVNGK, AIGLVIPELNGKLDGAAQR, VVSWYDNEMSYTAQLVR, VSAERDPEQIDWANDGVEIVLEATGFFATK, ALQDNFGVVEGLMoxTTIHAYTGDQMoxILDGPHR, AVANESYGYTEDPIVSSDVVGMoxSYGSLFDATQTK. |
| Gapdh-3 | VLDVDGK, VGINGFGR, TLEYFAK, FDGTVEVK, EGGFEVNGK, VVITAPGGSDVK, VLDVDGKQLVK, AIGLVIPELNGK, IQNVEGVEVTR, KVVITAPGGSDVK, NVTVDEVNAAMK, AGAANIVPNSTGAAK, RIQNVEGVEVTR, ARAGAANIVPNSTGAAK, INDLTDPVMLAHLLK, YDTTQGRFDGTVEVK, VPTPTGSVTELVVVLEK, FDGTVEVKEGGFEVNGK, AIGLVIPELNGKLDGAAQR, VVSWYDNEMoxSYTAQLVR, LDGAAQRVPTPTGSVTELVVVLEK, DPEQIDWANDGVEIVLEATGFFATK. |
| GroEL-1 | GTFNVVAVK, GVDILADTVK, NVTAGANPIGIR, EAIAQVAAVSSR, VGEYISEAMEK, VGNDGVITIEESK, GYLSQYMVTDSEK, SQIESATSEFDKEK, MVADLDNPYILITDK, DSTVIVEGAGNPEAIANR, ISNIQEILPLLESILK, TNDIAGDGTTTATVLTQAIVR, TNRPLLIIADDVDGEALPTLVLNK. |
| GroEL-2 | LSGGVAVIK, GTFNVVAVK, IEDALNATR, VGAATETELK, GVDILADTVK, NVTAGANPIGIR, EAIAQVAAVSSR, DATIEALGQASK, VGEYISEAMEK, GIEAAVATAVSALK, VGNDGVITIEESK, RGIEAAVATAVSALK, GYLSQYMVTDSEK, SQIESATSEFDKEK, SFGSPLITNDGVTIAK, EIELEDHFENMGAK, MVADLDNPYILITDK, DSTVIVEGAGNPEAIANR, ISNIQEILPLLESILK, MVADLDNPYILITDKK, TNDIAGDGTTTATVLTQAIVR, VTVDKDSTVIVEGAGNPEAIANR. |
| HPr | FASDITLEYK, SIMoxGVMoxSLGVGQGADVTISAEGADADDAIAAISETMEK. |
| Imdh | VGIGPGSICTTR, TLIAGNIATAEGAR. |
| Ldh-1 | DAAYTIINK, VIGSGTSLDSAR, GATYYGIAVALAR, AVGDALDLSHALAFTSPK, DTQNVQEAELIELFEGVR, YEDCADADLVVITAGAPQKPGETR. |
| Ldh-2 | DAAYTIINK, VIGSGTSLDSAR, GATYYGIAVALAR, AVGDALDLSHALAFTSPK, DTQNVQEAELIELFEGVR, AILDDENAVLPLSVFQEGQYGVK. |
| Pfk-1 | YPEFAQK, GIGGVAVGIR, KGIGGVAVGIR, FEEIVASIK, VTELGHIQR, TFVVEVMoxGR, ADLALSSLNR, MoxGAHAVELLK, MoxGAHAVELLKK, GIGGVAVGIRNEK, IAVLTSGGDAPGMoxNAAIR, AAGDTSDLRVTELGHIQR, IIVNNPHKADLALSSLNR, HGIEGVVVIGGDGSYHGAMoxR, HNIIVLAEGVMoxSADEFGEK, HNIIVLAEGVMoxSADEFGEKLK, MoxVENPILGTAEEGALFSLAEDGK. |
| Pfk-2 | GIEQLKK, YPEFAQK, GIGGVAVGIR, IIVNNPHK, GGTFLHSAR, KGIGGVAVGIR, FEEIVASIK, TFVVEVMGR, VTELGHIQR, ADLALSSLNR, MGAHAVELLK, RTFVVEVMGR, MGAHAVELLKK, EGQLKGIEQLK, GIGGVAVGIRNEK, YPEFAQKEGQLK, IAVLTSGGDAPGMNAAIR, AAGDTSDLRVTELGHIQR, IIVNNPHKADLALSSLNR, HGIEGVVVIGGDGSYHGAMR, HNIIVLAEGVMSADEFGEK, HNIIVLAEGVMoxSADEFGEKLK, MoxVENPILGTAEEGALFSLAEDGK. |
| Pgam | GEANVELTPELAFK, ESGAAIGLAFDGDSDR, LIAVDENGELVDGDK, FGGYVLSQHESEVPK, KLSELASEVTIYPQK, ISGEMoxLESALIAGLLSVGIHVYK, VMoxAEAPSHEEVDFYVDTIADVVR. |
| Pgk-1 | ITAALPTIK, FDEALTGAK, SLAPVAADLAAK, EANAFADYTEVK, AHASNVGISANVEK, SIIGGGDSAAAAINLGR. |
| Pgk-2 | LILPVDSK, VDFNVPVK, ITAALPTIK, YILEQGGR, FDEALTGAK, AILFSHLGR, LTVKDVDLK, VLPGLAALTEK, SLAPVAADLAAK, IGVIENLLEK, SIAKFDEALTGAK, AHASNVGISANVEK, VLIGGGMTYTFYK, SIIGGGDSAAAAINLGR, VSDKIGVIENLLEK, ITAALPTIKYILEQGGR, VDFNVPVKDGVITNDNR, DTEGEAVDPGFLGLDIGPK, AQGIEIGNSLVEEDKLDVAK, ADKFSWISTGGGASMELLEGK, YWASLGDGIFVNDAFGTAHR, NDPELGKYWASLGDGIFVNDAFGTAHR, EANAFADYTEVKDTEGEAVDPGFLGLDIGPK. |
| Pgk-3 | VLPGLAALTEK, IGVIENLLEK, VLIGGGMTYTFYK, SIIGGGDSAAAAINLGR, DTEGEAVDPGFLGLDIGPK. |
| Pgm-1 | IAPALK, EYYLGK, HYGGLTGK, VKLVFAR, GTQQAIDAGK, NVFVGAHGNSIR, LNVVKEYYLGK, DGKNVFVGAHGNSIR, ALPFWEDKIAPALK, ANLFTGWADVDLSEK, EAGIEFDQAYTSVLK, YASLDDSVIPDAENLK, AEAAEQFGDEQVHIWR, TTNLALEAADQLWVPVEK, NKAEAAEQFGDEQVHIWR, SYDVLPPAMoxPHDDEYSAHTDRR, ANLFTGWADVDLSEKGTQQAIDAGK, HLSDDEIMoxGVEIPNFPPLVFEFDEK. |
| Pgm-2 | HYGGLTGK, VKLVFAR, IAPALKDGK, GTQQAIDAGK, NVFVGAHGNSIR, LNVVKEYYLGK, GTQQAIDAGKLIK, DGKNVFVGAHGNSIR, ALPFWEDKIAPALK, ANLFTGWADVDLSEK, EAGIEFDQAYTSVLK, YASLDDSVIPDAENLK, AEAAEQFGDEQVHIWR, TTNLALEAADQLWVPVEK, NKAEAAEQFGDEQVHIWR, YASLDDSVIPDAENLKVTLER, SYDVLPPAMoxPHDDEYSAHTDR, HLSDDEIMGVEIPNFPPLVFEFDEK. |
| Ppp | NIITQSIGQK, AGQITAEEAER, MoxEIALLTDVGK, IHEVGQSDEYK, DWFAEHLEAENK, QLTSDHSLVNALLK, TNNQDFVNHFVNR, SGMoxPLIVLADGMoxGGHR, FANNAGGLDNITVALVYFDK, FANNAGGLDNITVALVYFDK, FANNAGGLDNITVALVYFDKEEEA, AGNIASEMoxAVTDLGAAWVDTQIDSVNGVR. |
| Pyk-1 | VGFLLDTK, IPFPALAER, GDMGIEVPFEMVPVYQK. |
| Pyk-2 | LVVTLTK, GVNIPNTK, VGFLLDTK, LAEEIAGQK, LNSDTFER, TELFEGDAK, IPFPALAER, LIEAGANTFR, QKGVNIPNTK, IVATLGPAVEIR, NAQTLLNEYGR, VGFLLDTKGPEIR, VKIVATLGPAVEIR, EFEVVVENDGIIAK, TELFEGDAKEYSYK, FNFSHGDHQEQGER, AICEETGNGHVQLFAK, FGLEQGINFIAISFVR, GVNIPNTKIPFPALAER, TEVMoxASAVKDATNSMDIK, GDMGIEVPFEMVPVYQK, VVITATNMoxLETMoxTEKPR, FGEDGYWGEKLDVEASAK, DDATREFEVVVENDGIIAK, YRPNADILALTFDELTER, AVEAGLVQSGDDIVIVAGVPVGEAVR, DNDDIRFGLEQGINFIAISFVR, IENQQGIDNLDEIIEAADGIMIAR, KAVEAGLVQSGDDIVIVAGVPVGEAVR. |
| Rmla-1 | GIILAGGSGTR. |
| Rmla-2 | GIILAGGSGTR, GELEITDVNK. |
| rS1 | DTDTVTYLVSK, SVVTVGEEIEVK. |
| Tig | AVEVITSTAK, TNLVIEAIAK, EAGIEVVAQPK, EVTDEDVDAR, SVNEFLGNMQR, DIDEEVETLDELK, DIDEEVETLDELKEK, GQDWTISAEVVTKPEVK, AGETVDVVVTFPEDYQAADLAGK, EGPAAEGDTVVIDFVGSVDGVEFDGGK. |
| Tpi | AFVEAVASK, KPFIAGNWK, DVVAADFGQEVADK, DYFHETDEDINK, EIGTDYVVIGHSER, DYFHETDEDINKK, LPSSDLVEAGIAAPAVDLTAVLAAAK, VAAQNCYFENSGAFTGETSPQVLK, AIFANGMoxLPIICCGESLETYEAGK. |

| **Table B. Non-phosphorylated protein species** | |
| --- | --- |
| ABC | ANHLLEDLK, VQLILVMoxSR, IVGLLGPNGSGK, HDNTIDHIFR, KHDNTIDHIFR, STSSLLRLEHVQK, ANHLLEDLKLDEK, TEALSGVNLDIQPGR, DGEILLQEDANELR, DGEILLQEDANELRR, VVSYLPDTTYLGDNMoxK, ADLYVLDEPIGGVDPAAR, KVVSYLPDTTYLGDNMoxK, KADLYVLDEPIGGVDPAAR, NYGKTEALSGVNLDIQPGR, EVFAMoxFQDFYSDFDQAK, IKEVFAMoxFQDFYSDFDQAK, LINGLLQPTSGQVLIHGEKPSPK. |
| aced-1 | LVQDGFK, GITQTLAR, AVAELSATK, RLVQDGFK, TFAVVADVSK, QAEVATAFQK, DDEWGMoxQTFAK, VGLLDYNAETVEK, TPMoxMoxFDIAHEVGK, VAIVTGAGQGIGFAIAK, NAGKDDEWGMoxQTFAK, DLADSGITVNAYAPGIVK, TFAINVGGVIWGSQAAQAQFK, IINATSQAGVVGNPNLTVYGGTK, VVDHFGDLNVVVNNAGVAPTTPLDTITEEQFTR. |
| aced-2 | DITLKR, LVQDGFK, RLVQDGFK, TFAVVADVSK, QAEVATAFQK, DDEWGMQTFAK, VGLLDYNAETVEK, TPMoxMoxFDIAHEVGK, VAIVTGAGQGIGFAIAK, NAGKDDEWGMoxQTFAK, VAIVTGAGQGIGFAIAKR, SKVAIVTGAGQGIGFAIAK, DLADSGITVNAYAPGIVK, TFAVVADVSKQAEVATAFQK, TFAINVGGVIWGSQAAQAQFK, IINATSQAGVVGNPNLTVYGGTK, RLSEPEDVAAAVSFLAGPDSDYITGQTIIVDGGMQFH. |
| aced-3 | AVAELSATK, RLVQDGFK, TFAVVADVSK, QAEVATAFQK, DDEWGMQTFAK, VGLLDYNAETVEK, TPMoxMoxFDIAHEVGK, VAIVTGAGQGIGFAIAK, NAGKDDEWGMoxQTFAK, SKVAIVTGAGQGIGFAIAK, DLADSGITVNAYAPGIVK, TFAINVGGVIWGSQAAQAQFK, IINATSQAGVVGNPNLTVYGGTK. |
| adh | ILGHEGIGIVK, VAFNQAVDSVR, TNGGAHSAVVTAVSK, TVLDGIQVIGSLVGTR, VFNAHVIAVDINNDK, AVVVNPESTGVEIIQNK, VVAVGLPSEMoxMoxDLSIVK, DLEEAFQFGAEGLVVPVVEK, EVGADIVINGHEVDDVPGLIK, VPEGLDPAQASSITCAGVTTYK, RPVEDAVDVFDEMoxEAGTIQGR, NAGYSVDGGMoxAEQCIVTADYAVK, EAQLQPGQWIVLYGAGGLGNLAVQYAK. |
| adk | VAHISTGDMFR, ETGFLLDGYPR, TIDQAHALDAILK, AAMANQTEMGVLAK, RLDVNIAQGEPILSHYR, VFNPPANYNEEDYYQR. |
| alad | MoxLIGIPK, VTTHTEPVYEK, MoxAVQIGAHFLTK, VALTPAGVHSLVGK, VTIIGGGVVGTHAAR, EADVVIGAVLIPGAK, QEGGSGVLLGGVPGVPK, GKVTIIGGGVVGTHAAR, IVPTAAEAWAAELVVK, IALGLGAQVTILDISAK, QEGGSGVLLGGVPGVPKGK, HGVLHYAVANIPGAVAR, EADVVIGAVLIPGAKAPK, IALGLGAQVTILDISAKR, DLEGQLPLLVPMoxSEVAGR, TSTIALTNVTLPYVEALAEK, GHEVLIETNAGLGSGFADADYEK, QMoxRPGSVIVDVAVDQGGVIETADR, GHEVLIETNAGLGSGFADADYEKQGAK. |
| aprt | ATIEMIER, VISADYEK, EIVQYATDK, IDMVVGPEAR, EVIGEYDYK, EIVQYATDKK, KIDMVVGPEAR, DYIATIENYPK, VLIVDDLLATGGTVK, DISPLMADGNAYSYAVR, LGGIVAGCAFLIELDELK, GFIVGCPVAFELGIGFAPVR. |
| clpp | TVEQIHADAER, TLEQILADNSGK, KTLEQILADNSGK. |
| daph | TALEDALR, MNAQEIIK, TYVVEQDAR, NSAVPLLDKR, NSHVGAGAVLAGVIEPASAEPVR. |
| dera | IGASSGVAIMK, LAAEGLSGSDVK, SYDDAQAFIK, DIQAVVEASGEK, VIIETCLLTDQEK, VCTVIGFPLGATTPEVK, EYDFASVCVNPTWVK, TSTGFSTGGATVEDVALMR. |
| DnaK-4 | Deduced from adjacent DnaK 1-2. |
| DnaK-5 | FQLTDIPAAPR, NEVDQAIFATEK, EQTIVIQSNSGLTDEEIDR, SVNPDEVVAMGAAIQGGVITGDVK. |
| DnaK-6 | IPAVVEAVK, GYAEEYLGEK, AVITVPAYFNDAQR, IVNEPTAAALAYGLDK, EYTPQEISAMILQYLK, IIGIDLGTTNSAVAVLEGTESK. |
| Ef-G | VYSGVLQSGSYVLNTSK. |
| Ef-Ts | ALVETDGDIEK, NAQFVDLVNATAK, IGVISVIEGGDEALAK, TDAQHFGAYQHNGGR, AQLTDAVVAQAEEDIK. |
| Ef-Tu-5 | VNDEIEIVGIK, TTLTAAITTVLAR, QLDEGLAGDNVGVLLR, DTDKPLLLPVEDVFSITGR, DLLSEYDFPGDDLPVIQGSALK, YEDIIMoxDLMoxNTVDEYIPEPER. |
| Ef-Tu-6 | AVVTGVEMFR, TVGSGMVTEIEA, VNDEIEIVGIK, QLDEGLAGDNVGVLLR, VNDEIEIVGIKEEIQK, DTDKPLLLPVEDVFSITGR. |
| Ef-Tu-7 | AVVTGVEMFR, FKGEVYILTK, TVGSGMVTEIEA, VNDEIEIVGIK, QLDEGLAGDNVGVLLR, VNDEIEIVGIKEEIQK. |
| Eno-11 | AMoxIALDGTPNK, IEDQLGEVAQYK, LGANAILGVSIAVAR, GLETAVGDEGGFAPR, TAAEQIDYLEELVNK, GMoxVPSGASTGEHEAVELR, DVFIGFDCASSEFYDK, GNPTLEVEVYTESGAFGR, VNQIGTLTETFDAIEMoxAK, AVDNVNNVIAEAIIGYDVR, VQLVGDDFFVTNTDYLSR, DVFIGFDCASSEFYDKER, SGETEDSTIADIAVATNAGQIK, AAADYLEIPLYSYLGGFNTK, FEGTEDGVETIIAAIEAAGYVPGK. |
| Eno-12 | SIITDVYAR, GLQAFYNLK, EGCANSILIK, AMIALDGTPNK, EAGYTAVVSHR, IEDQLGEVAQYK, LGANAILGVSIAVAR, GLETAVGDEGGFAPR, DVFIGFDCASSEFYDK, VNQIGTLTETFDAIEMAK, AVDNVNNVIAEAIIGYDVR, VQLVGDDFFVTNTDYLSR, SGETEDSTIADIAVATNAGQIK, AAADYLEIPLYSYLGGFNTK. |
| Eno-13 | GLQAFYNLK, EGCANSILIK, EAGYTAVVSHR, IEDQLGEVAQYK, DVFIGFDCASSEFYDK, VNQIGTLTETFDAIEMAK, VQLVGDDFFVTNTDYLSR, SGETEDSTIADIAVATNAGQIK. |
| Eno-14 | FEGEGAAVR, GLQAFYNLK, EGCANSILIK, EAGYTAVVSHR, IEDQLGEVAQYK, GLETAVGDEGGFAPR, TAAEQIDYLEELVNK, VNQIGTLTETFDAIEMAK, VQLVGDDFFVTNTDYLSR, SGETEDSTIADIAVATNAGQIK, AAADYLEIPLYSYLGGFNTK. |
| Eno-15 | SIITDVYAR, AMIALDGTPNK, YGAEIFHALK, LGANAILGVSIAVAR, GLETAVGDEGGFAPR, GNPTLEVEVYTESGAFGR, AVDNVNNVIAEAIIGYDVR, AAADYLEIPLYSYLGGFNTK, FEGTEDGVETIIAAIEAAGYVPGK. |
| Eno-16 | SIITDVYAR, AMIALDGTPNK, YGAEIFHALK, LGANAILGVSIAVAR, GLETAVGDEGGFAPR, GMVPSGASTGEHEAVELR, GNPTLEVEVYTESGAFGR, AVDNVNNVIAEAIIGYDVR, AAADYLEIPLYSYLGGFNTK. |
| Eno-17 | SIITDVYAR, AMIALDGTPNK, YGAEIFHALK, LGANAILGVSIAVAR, GLETAVGDEGGFAPR, GMVPSGASTGEHEAVELR, GNPTLEVEVYTESGAFGR, AVDNVNNVIAEAIIGYDVR, AAADYLEIPLYSYLGGFNTK. |
| Eno-18 | GLQAFYNLK, EAGYTAVVSHR, IEDQLGEVAQYK, VQLVGDDFFVTNTDYLSR. |
| Eno-19 | YAEIVDSDAVAK, TVTIQEVGETEK, VSNESPIGQALIGK, GDIATVETPVGSYDVK, SYGDLSENSEYEAAK, KGDIATVETPVGSYDVK, YAEIVDSDAVAKDEVGIGK, TVTIQEVGETEKEVYIIVGSAGADAFAGK, SYGDLSENSEYEAAKDEQAFVEGQISSLETK. |
| Eno-20 | GLQAFYNLK, EGCANSILIK, EAGYTAVVSHR, IEDQLGEVAQYK, VNQIGTLTETFDAIEMAK, VQLVGDDFFVTNTDYLSR, SGETEDSTIADIAVATNAGQIK. |
| Eno-21 | GLQAFYNLK, EGCANSILIK, EAGYTAVVSHR, IEDQLGEVAQYK, VNQIGTLTETFDAIEMAK,, VQLVGDDFFVTNTDYLSR, SGETEDSTIADIAVATNAGQIK. |
| Eno-22 | SIITDVYAR, AMIALDGTPNK, IEDQLGEVAQYK, LGANAILGVSIAVAR, GMVPSGASTGEHEAVELR, GNPTLEVEVYTESGAFGR, AVDNVNNVIAEAIIGYDVR, SGETEDSTIADIAVATNAGQIK, AAADYLEIPLYSYLGGFNTK. |
| Eno-23 | SIITDVYAR, AMIALDGTPNK, LGANAILGVSIAVAR, GMVPSGASTGEHEAVELR, GNPTLEVEVYTESGAFGR, AVDNVNNVIAEAIIGYDVR. |
| Eno-24 | SIITDVYAR, AMIALDGTPNK, YGAEIFHALK, YGAEIFHALKK, LGANAILGVSIAVAR, GMVPSGASTGEHEAVELR, GNPTLEVEVYTESGAFGR, AVDNVNNVIAEAIIGYDVR, AAADYLEIPLYSYLGGFNTK. |
| Fba-1 | Deduced from adjacent fba 2-3. |
| Fba-2 | IDVFGSEGKA, GELAPIEDAK, APVLIQTSMoxGAAK, EYDANEAEYDK, KAPVLIQTSMoxGAAK, EYDANEAEYDKK, FLKPGFEAITAAVEER, VNVNTECQIAFANATR, KFLKPGFEAITAAVEER, GISVEAEVGTIGGEEDGIIGK, DNGYAVGGFNTNNLEWTQAILR, KLTEALPGFPIVLHGGSGIPDDQIQAAIK. |
| Fba-3 | IDVFGSEGK, IDVFGSEGKA, GELAPIEDAK, APVLIQTSMoxGAAK, EYDANEAEYDK, AIVSAEKFVQAAR, KAPVLIQTSMoxGAAK, EYDANEAEYDKK, FLKPGFEAITAAVEER, VNVNTECQIAFANATR, KFLKPGFEAITAAVEER, GISVEAEVGTIGGEEDGIIGK, DNGYAVGGFNTNNLEWTQAILR, GISVEAEVGTIGGEEDGIIGKGELAPIEDAK. |
| Fba-4 | Deduced from adjacent fba 2-3. |
| Fba-5 | APVLIQTSMGAAK, KAPVLIQTSMGAAK, GISVEAEVGTIGGEEDGIIGK. |
| Gapdh-4 | IQNVEGVEVTR, VVSWYDNEMSYTAQLVR. |
| Gapdh-5 | FDGTVEVK, AIGLVIPELNGK, IQNVEGVEVTR, VVSWYDNEMSYTAQLVR. |
| Gapdh-6 | AIGLVIPELNGK, IQNVEGVEVTR. |
| Gapdh-7 | VGINGFGR, FDGTVEVK, AIGLVIPELNGK, IQNVEGVEVTR, VVSWYDNEMSYTAQLVR. |
| Gapdh-8 | AIGLVIPELNGK, VVSWYDNEMSYTAQLVR. |
| glya | AVMAAQGSILTNK, GGLILTNDEDLAK, ELTDAFPLYEGLN, ALENAENEAVLNQVR, VAQNLLDEVNITLNK, QQHNIELIASENVVSK, VISDGTENHLFLVDVTK, YYGGTDVVDVIESLAIER. |
| greA | YAEIVDSDAVAK, TVTIQEVGETEK, VSNESPIGQALIGK, GDIATVETPVGSYDVK, SYGDLSENSEYEAAK, KGDIATVETPVGSYDVK, YAEIVDSDAVAKDEVGIGK, TVTIQEVGETEKEVYIIVGSAGADAFAGK, SYGDLSENSEYEAAKDEQAFVEGQISSLETK. |
| GroEL-3 | GTFNVVAVK, VGAATETELK, NVTAGANPIGIR, EAIAQVAAVSSR, VGEYISEAMEK, GIEAAVATAVSALK, VGNDGVITIEESK, GYLSQYMVTDSEK, SQIESATSEFDKEK, DSTVIVEGAGNPEAIANR, TNDIAGDGTTTATVLTQAIVR, VTVDKDSTVIVEGAGNPEAIANR, TNRPLLIIADDVDGEALPTLVLNK. |
| GroES | TLTGELVAPSVK. |
| grpE | SELELANER, AILPSLDNLER, ALAVEGLTDDVKK. |
| Hyd | GPFATIK, GGTPHLDR, TVANFIALSK, LVLFPEQAPK, DGYYDGVIFHR, ELNFPQVEIENNK, FEDEFSPELYNIR, HTVFGQLLDEQSYQVLDK, IIQDFMIQGGDPTGTGMGGESIYGER, ISQVETGAMDRPVEDVIIETIEVEDE. |
| Imdh-2 | VGIGPGSICTTR, YFQGSVNEANK, TLIAGNIATAEGAR, AEALFEAGADAIVIDTAHGHSAGVLR. |
| Ldh-3 | DAAYTIINK, DAAYTIINKK, VIGSGTSLDSAR, ELQAIIDEAWK, GATYYGIAVALAR, AVGDALDLSHALAFTSPK, DTQNVQEAELIELFEGVR, YEDCADADLVVITAGAPQKPGETR. |
| murd | VILIAGGLDR, VDGAYLDGDVLTFR, AADQAGVSYLDATDVR, LQYVGQVNEVAFYNDSK. |
| nade | FGDGGADILPLYR, QLLAALGADPALYEK, SYVLGISGGQDSTLAGR. |
| pept | DFETDAFENR, FEYVSLETMER, VGFGPDEEIGIGADK, GQTLITTDGTTLLGADDK. |
| Pgk-4 | Deduced from adjacent pgk 1-2. |
| Pgk-5 | Deduced from adjacent pgk 1-2. |
| Pgk-6 | VDFNVPVK, SLAPVAADLAAK, SIIGGGDSAAAAINLGR. |
| Pgk-7 | VDFNVPVK, ITAALPTIK, AILFSHLGR, SLAPVAADLAAK, IGVIENLLEK, AHASNVGISANVEK, VLIGGGMTYTFYK. |
| Pgk-8 | LILPVDSK, FDEALTGAK, VLPGLAALTEK, IGVIENLLEK, EANAFADYTEVK, VLIGGGMTYTFYK, SIIGGGDSAAAAINLGR, FSWISTGGGASMELLEGK, DTEGEAVDPGFLGLDIGPK, AQGIEIGNSLVEEDKLDVAK, TVVWNGPMGVFENPDFQAGTIGVMDAIVK. |
| Pgk-9 | VDFNVPVK, ITAALPTIK, YILEQGGR, AILFSHLGR, SLAPVAADLAAK, AHASNVGISANVEK, VDFNVPVKDGVITNDNR, YWASLGDGIFVNDAFGTAHR, GAELEAAINALEDGQVLLVENTR. |
| pyrb | DVEIADHLVEAPK. |
| rL1 | AGIVQAIIGK, AYSVEEAVALAK, AAGADFVGEDDLVAK. |
| rL4-1 | VLAALSIDTK, LLVTQAAISK, VATATTASVLDIVNSDK, VLVILEEGNEFAALSAR. |
| rL4-2 | VLAALSIDTK, LLVTQAAISK, FVAVDSLSFTAPK, VATATTASVLDIVNSDK. |
| rL10 | NVALAVK, ILNDFAK, NADALEIK, GGAIEGAVASK, AVADNKEDAA, AAASIVVVDAR, KAELVDVVAEK, ELRGSEVEYK, GLTVEQDTVLR, EEILALATLPNR, MoxKAAASIVVVDAR, EGLLSMoxLLSVLQAPVR, NADALEIKGGAIEGAVASK, GGAIEGAVASKEEILALATLPNR, AGLEDLASVFVGPSAVAFSNEDVIAPAK. |
| rL17 | LFSELAPR, NEIASENYDEATEK. |
| rmlb | ANLEEILGDR, FHHVSTDEVYGDLPLR, ATISNCSNNYGPYQHIEK. |
| rrf | ANASLLDR, VLLVTPFDK, AKEITEDELK, LVIPALTEETR, LVIPALTEETRR, VLLVTPFDKSSLK, AINASDLGITPASDGSVIR, ISVEYYGVETPLNQIASITIPEAR. |
| rpo | GYGTTLGNSLR, EDVMQIILNIK, AGINTVYDLTEK, ILTEHLDLFTDLTEVAK, DDAPVGTLAVDSIYTPVTK, RDDAPVGTLAVDSIYTPVTK, LTLEIMoxTNGTIIPEDALGLSAR. |
| rpoZ | YSLVILEAK, MMLKPSIDTLLDK, MMLKPSIDTLLDKVPSK, ALEEIESGNVTIHPDPEGK, ALEEIESGNVTIHPDPEGKR. |
| rS2 | MADAIIEGR, AGQYYINHR, FLGGIEEMPR, YADQAYDFIR, MEEDGTFEVLPK, NGIHVIDLQQTVK, DAAANDAIILFVGTK, WLGGTLTNWSTIQK. |
| rS5-1 | FAALVVVGDR, SLGSNTPINIVR, AVIELAGVADVTSK, VLLKPAVEGSGVAAGGAVR. |
| rS5-2 | FAALVVVGDR, SLGSNTPINIVR, AVIELAGVADVTSK, VLLKPAVEGSGVAAGGAVR. |
| rS8 | NVEFIEDDKQGIIR, VLNGLGIAILSTSEGLLTDK. |
| rS17 | TITVVVETK. |
| sed | EVAEFDLLNTFK, NITPEDVAVAQELGYVVK, GQVLHLAEIFNAEDVSFK. |
| sepF | CIDYLDGAK, RCIDYLDGAK, LPDGVHSEFDFDMK, QQELAQNHSVATSEK, FIEYFTEDGDDVQVAESR, LVSSSSQHVTNTPSSNENITR. |
| sod | VNELYAAAK, NVRPDYIK, HPEIGEDLEK, AFFSVINWNK, FGSGWAWLVVNK, LLADVESIPADIR, HHNTYVTNVNVALAK, LEVVSTANQDTPISEGK. |
| Sys-1 | NTVSAEIAQAK, NLDATLAELDAK, LLHTLNGSGLAVGR, VVALCTGDMGFSAAK, FAKPEESYEELEK, EISSCSNTEDFQAR, ELPIYFTAMSPSFR. |
| Sys-2 | NLDATLAELDAK. |
| tox | KTLADFAGK, TTFLGNPVTFTGK, DYAVLINEWHLLAR, VLSIIPSIDTGVCSTQTR, QLQVGDTAHDFSLTATDLSK, WCAAEGIENAVMLSDYFDHSFGR. |
| Tpi-2 | AFVEAVASK, KPFIAGNWK, DVVAADFGQEVADK, DYFHETDEDINK, EIGTDYVVIGHSER, DYFHETDEDINKK, LPSSDLVEAGIAAPAVDLTAVLAAAK, VAAQNCYFENSGAFTGETSPQVLK, AIFANGMLPIICCGESLETYEAGK. |
| pxk | ELYDLGAK, SPLAAVQFSK, AVDVYYDGR, DFVYGAIQR, ENHDLEVSALR, SDQYGVVQYEK, QHADIPVVLDPVLVCK, IGLLPNLQIAEQALAFVK, GFEVIPVEEEVFAQQLASLK, FFPYVSIITPNLAEAQLLTQK. |
| pox | FIQVDIDPYK, LFLEEEGLQSR, SFIAPALNEVEIDK. |
| Pyk-3 | Deduced from adjacent pyk 1-2. |
| Pyk-4 | Deduced from adjacent pyk 1-2. |
| xprt | GLVEIIR, LLEDDGQR, DGNVLGENILK, QAGASVEAIGIVIEK, ILRDGNVLGENILK, LLEDDGQRVVSLAR, VLVIDDFLANGQAAK, VDSFLTHQVDFTLMoxK, EIGQVFADIFHESGITK, NITMoxTEGILTAEVYSFTK, FLSPSDKVLVIDDFLANGQAAK, VVTIEASGIAPAVYTAEALGVPMIFAK. |
| y1595 | QLDQQQQLTAK. |
| y454 | GNTDETFTEGR, LVDVLEDDEDVQK, NGGNMGASGSVSYLFDNK, YEGFGPNGSMLIVDTLTSNVNR. |
